# Effect of a Lifestyle Intervention on Cardiometabolic Health Among Emerging Adults: A Randomized Clinical Trial

**DOI:** 10.1001/jamanetworkopen.2022.31903

**Published:** 2022-09-19

**Authors:** Jessica Gokee LaRose, Tricia M. Leahey, Autumn Lanoye, Melanie K. Bean, Joseph L. Fava, Deborah F. Tate, Ronald K. Evans, Edmond P. Wickham, Megan M. Henderson

**Affiliations:** 1 Department of Health Behavior and Policy, School of Medicine, Virginia Commonwealth University, Richmond; 2 Department of Allied Health Sciences, University of Connecticut, Storrs; 3 Massey Cancer Center, Virginia Commonwealth University, Richmond; 4 Department of Pediatrics, School of Medicine, Virginia Commonwealth University, Richmond; 5 Children’s Hospital of Richmond at Virginia Commonwealth University, Richmond; 6 Centers for Behavioral and Preventive Medicine, The Miriam Hospital, Providence, Rhode Island; 7 Department of Nutrition, University of North Carolina at Chapel Hill, Chapel Hill; 8 Lineberger Comprehensive Cancer Center, University of North Carolina at Chapel Hill, Chapel Hill; 9 Department of Kinesiology and Health Sciences, Virginia Commonwealth University, Richmond; 10 Department of Internal Medicine, School of Medicine, Virginia Commonwealth University, Richmond

## Abstract

**Importance:**

The prevalence of obesity has increased substantially among emerging adults, yet no previous large-scale behavioral weight loss trials have been conducted among this age group.

**Objective:**

To test the effect of 2 theory-based motivational enhancements on weight loss within a primarily digital lifestyle intervention designed for emerging adults.

**Design, Setting, and Participants:**

In this randomized clinical trial conducted at an academic medical research center, 382 participants aged 18 to 25 years with a body mass index (BMI; calculated as weight in kilograms divided by height in meters squared) of 25 to 45 were enrolled between February 2, 2016, and February 6, 2019. Data collection was completed February 8, 2020. Analysis was performed on an intention-to-treat basis.

**Interventions:**

Participants were randomized to 1 of 3 groups: developmentally adapted behavioral weight loss (aBWL), aBWL plus behavioral economics (aBWL + BE), or aBWL plus self-determination theory (aBWL + SDT). All groups received a 6-month intervention with 1 group session, 1 individual session, and a digital platform (digital tools for self-monitoring, weekly lessons, tailored feedback, text messages, and optional social media). The aBWL + BE group received modest financial incentives for self-monitoring and weight loss; the aBWL + SDT group received optional experiential classes. Coaching and message framing varied by group.

**Main Outcomes and Measures:**

The primary outcome was mean (SE) weight change (in kilograms) at 6 months. Secondary outcomes included proportion of participants achieving weight loss of 5% or more, percentage weight change, waist circumference, body composition, and blood pressure.

**Results:**

Among the 382 participants (mean [SD] age, 21.9 [2.2] years), 316 (82.7%) were female, mean (SD) BMI was 33.5 (4.9), 222 (58.1%) were of underrepresented race and/or ethnicity, and 320 (83.8%) were retained at the primary end point. There was a significant time effect for mean (SE) weight loss (−3.22 [0.55] kg in the aBWL group; −3.47 [0.55] kg in the aBWL + BE group; and −3.40 [0.53] kg in the aBWL + SDT group; all *P* < .001), but no between-group differences were observed (aBWL vs aBWL + BE: difference, −0.25 kg [95% CI, −1.79 to 1.29 kg]; *P* = .75; aBWL vs aBWL + SDT: difference, −0.18 kg [95% CI, −1.67 to 1.31 kg]; *P* = .81; and aBWL + SDT vs aBWL + BE: difference, 0.07 kg [95% CI, −1.45 to 1.59 kg]; *P* = .93). The proportion of participants achieving a weight loss of 5% or more was 40.0% in the aBWL group (50 of 125), 39.8% in the aBWL + BE group (51 of 128), and 44.2% in the aBWL + SDT group (57 of 129), which was not statistically different across groups (aBWL vs aBWL + BE, *P* = .89; aBWL vs aBWL + SDT, *P* = .45; aBWL + SDT vs aBWL + BE, *P* = .54). Parallel findings were observed for all secondary outcomes—clinically and statistically significant improvements with no differences between groups.

**Conclusions and Relevance:**

In this randomized clinical trial, all interventions produced clinically significant benefit, but neither of the motivational enhancements promoted greater reductions in adiposity compared with the developmentally adapted standard group. Continued efforts are needed to optimize lifestyle interventions for this high-risk population and determine which intervention works best for specific individuals based on sociodemographic and/or psychosocial characteristics.

**Trial Registration:**

ClinicalTrials.gov Identifier: NCT02736981

## Key Points

**Question:**

What is the effect of enhancements that target intrinsic vs extrinsic motivation on weight loss outcomes in a primarily digital lifestyle intervention designed specifically for emerging adults?

**Findings:**

In this randomized clinical trial of 3 adapted behavioral interventions that included 382 emerging adults with overweight or obesity, all 3 interventions led to statistically and clinically significant weight losses in excess of 3% of initial body weight. However, there were no significant differences between the intervention groups.

**Meaning:**

All 3 adapted behavioral interventions worked well on average and could be used to promote weight management during this vulnerable developmental period, but more work is needed to understand which intervention works best for specific individuals based on sociodemographic and/or psychosocial characteristics.

## Teaser

This randomized clinical trial tests the effect of 2 theory-based motivational enhancements on weight loss within a primarily digital lifestyle intervention designed for emerging adults.

## Introduction

Obesity is a public health challenge. Lifestyle management is the recommended first-line treatment,^1^ but there is a dearth of evidence for treatment of obesity for emerging adults. Emerging adulthood, often defined as 18 to 25 years of age, is a distinct developmental period that occurs across educational and socioeconomic strata—these years represent a time of instability, identity formation, and multiple life transitions.^2^ Emerging adulthood is also marked by considerable risk for obesity.^3^ During the last 4 decades, the prevalence of obesity among emerging adults has increased from 6.2% to 32.7% and more than half of emerging adults currently meet the criteria for overweight or obesity as defined by body mass index (BMI; calculated as weight in kilograms divided by height in meters squared).^3^

The urgency of promoting cardiometabolic health during the broader period of young adulthood (18-35 years) has been emphasized by the National Institutes of Health (NIH)^4,5^ and evidence underscores the importance of weight management to mitigate cardiometabolic risk.^4,5,6,7^ Furthermore, the need to use technology to reach young adults has been well documented.^4,8,9^ However, e-Health weight loss trials targeting young adults have produced modest outcomes^10,11,12,13,14^ and researchers have cautioned against overreliance on technology alone to promote clinically significant weight loss among this population.^12,14^ Moreover, only a handful of small pilot trials have focused on weight loss for emerging adults specifically,^15,16,17^ despite their unique developmental considerations,^2^ the recent increase in obesity prevalence,^3^ elevated vulnerability to excessive weight gain when untreated,^18,19^ and calls from researchers to focus specifically on this age group.^3,8,17,20^ Although the NIH invested in the EARLY (Early Adult Reduction of Weight Through Lifestyle Intervention) trials,^21^ a consortium of studies focused on young adults,^4,21^ none of those trials focused specifically on emerging adults.

Effective intervention during emerging adulthood has the potential to mitigate cardiometabolic risk and decrease the cumulative burden of disease over the lifespan. To that end and grounded in extensive formative^8^ and pilot work,^15,16,22^ we adapted standard behavioral weight management to meet the developmental, psychosocial, and pragmatic needs of emerging adults. The intervention incorporated evidence-based behavior change techniques and was delivered via hybrid format, pairing a multicomponent digital platform with minimal in-person contact. Formative data highlighted 2 key drivers of motivation that could be used to promote engagement—autonomy and money.^8^ Guided by these formative data,^8^ extant evidence,^15,22,23^ and theory,^23,24,25,26,27,28,29^ we developed 2 motivational enhancements to integrate with this new adapted standard program. The first was grounded in behavioral economics (BE)^23,24,25,26^ and used modest financial incentives tied to self-monitoring and weight loss. The second approach was rooted in self-determination theory (SDT)^27,28,29^ and sought to enhance values-driven lifestyle change and support autonomy, competence, and relatedness. We report findings from a comparative efficacy trial^30^ testing these lifestyle interventions on weight change at 6 months (primary outcome), as well as secondary cardiometabolic outcomes, including the proportion of participants achieving a weight loss of 5% or more, BMI, waist circumference, body fat, and blood pressure. Based on pilot evidence,^15,22^ we hypothesized that both motivational enhancement groups would experience greater weight loss compared with the group receiving only the adapted standard after treatment (6 months). Given previous findings,^22,23^ we also hypothesized that the BE group would experience greater weight loss than the SDT group. Parallel hypotheses were specified for all secondary outcomes.

## Methods

### Study Design and Participants

**Figure 1.**
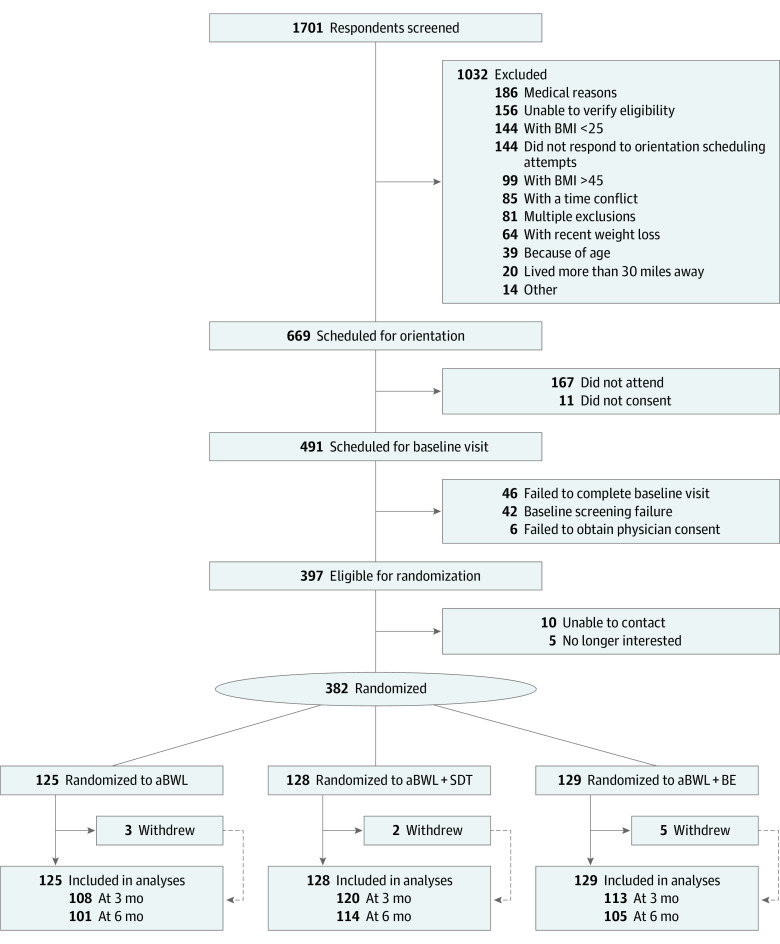
CONSORT Flow Diagram aBWL indicates developmentally adapted behavioral weight loss; BE, behavioral economics; BMI, body mass index (calculated as weight in kilograms divided by height in meters squared); and SDT, self-determination theory.

The Richmond Emerging Adults Choosing Health (REACH) trial was a 3-group randomized clinical trial designed to test the comparative efficacy of 3 behavioral weight loss (BWL) programs for emerging adults. A sample size of 381 was determined using an a priori power calculation; using estimates of variability from our pilot trials, we had 90% power to detect a clinically meaningful difference between groups, allowing for a 15% attrition rate and adjusting for 3 planned group comparisons.^30^ The single-site trial was conducted in Richmond, Virginia, and funded by the National Institutes of Diabetes and Digestive and Kidney Diseases. Enrollment occurred from February 2, 2016, to February 5, 2019; the trial was closed to accrual after reaching the predetermined sample size, and follow-up assessments continued through February 8, 2020 (Figure 1). The trial protocol is available in Supplement 1. The study followed the Consolidated Standards of Reporting Trials (CONSORT) reporting guideline for randomized clinical trials. Participants provided written informed consent and procedures were approved by the institutional review board at Virginia Commonwealth University.

Study design and methods have been described elsewhere.^30^ Participants were recruited using a multimethod approach spanning digital, social media, radio, print, and in-person outlets. Individuals were directed to a recruitment website where they could watch a brief video, learn key study details, and complete a secure prescreening questionnaire. Those appearing to be eligible were invited to an orientation session followed by an informed consent process, culminating in written consent.^30^ The REACH trial enrolled individuals aged 18 to 25 years with a BMI of 25 to 45. Exclusion criteria^30^ centered on safety (eg, ability to engage in physical activity, no history of anorexia or bulimia or recent compensatory behaviors), with some exclusions for confounding (eg, taking weight loss medications) and practical considerations that would interfere with potential intervention benefit (eg, lack of consistent internet access).

### Randomization and Masking

After baseline assessments, the statistician (J.L.F.) allocated eligible participants to either the adapted BWL (aBWL), aBWL + BE, or aBWL + SDT group using a variably sized, permuted block scheme, which was stratified based on sex assigned at birth (male or female), baseline BMI (<35 or >35), and race and ethnicity (non-Hispanic White or underrepresented race and ethnicity [given what groups are historically underrepresented in or excluded from BWL trials, this category included Black or African American, Latinx, American Indian or Native American, and Asian]). Randomization assignment was revealed to participants and interventionists at the group kickoff. The principal investigator (J.G.L.) and all assessors were masked until after the final data collection visit.

### Intervention

The conceptual model and interventions have been described in detail.^30^ Participants received the same core intervention grounded in behavioral self-regulation^31,32,33^ that was adapted to meet the practical, developmental, and psychosocial needs of emerging adults, including changes to content, delivery mode, and increased personalization.^8,30^ The core intervention included 1 group kickoff session (approximately 90 minutes) and 1 individual session to personalize goals (approximately 30 minutes), followed by a digital platform. Dietary goals were personalized based on baseline resting metabolic rate (assessed via indirect calorimetry) to yield weight losses of 0.45 to 0.9 kg per week. Physical activity progressions were determined based on baseline activity levels (measured via accelerometry); goals increased gradually toward 250 minutes per week of moderate-to-vigorous physical activity. Participants received digital tools (self-monitoring app and wireless scale) to facilitate self-monitoring and weekly tailored, written feedback via email on goal progress. In addition, participants received weekly video and pdf content, weekly automated text messages, and access to an optional private Facebook group to facilitate social support, moderated by the interventionist for that group.^30^

The motivational enhancements have been previously detailed.^30^ In addition to the core lifestyle intervention, the aBWL + BE intervention included small financial incentives. Guaranteed incentives were linked to behavior: for each week that participants self-monitored 4 days or more of weight and diet, they received a variable incentive ranging from $1 to $10. The maximum amount paid over 6 months was $90, or a mean of $3.75 per week. Lottery-based (not guaranteed) incentives were linked to outcome: participants who lost 5% to 10% of weight were entered into a raffle for $50; those who lost 10% or more of weight were entered into a raffle for $100 at 3 and 6 months. Feedback and text messages were directive and occasionally incorporated loss aversion messaging (eg, “Don’t miss out on your cash—be sure you self-monitor at least 4/7 days this week!”) and regret aversion messaging (eg, “The payout was $10 this week—don’t miss out on any more of your cash!”),^30^ which was designed to amplify the effects of the financial incentives.^23^ In contrast, the aBWL + SDT intervention emphasized intrinsic motivation and values-driven behavior change. Feedback was autonomy supportive and based on progress toward goals, but framed to underscore competence (ie, skill development, sense of effectiveness), relatedness (ie, connectedness, sense of belonging), and autonomy (ie, sense of volition and choice).^30^ Furthermore, feedback highlighted ways in which goal progress was aligned with participants’ values. Participants in the aBWL + SDT group also received access to 24 experiential group classes (eg, circuit training, cooking) offered through community partners. Classes were optional and designed to build competence and relatedness; no new content was taught.

### Intervention Fidelity

Interventionists were masked to hypotheses, played no role in assessments, and received standardized, group-specific training. Audio recordings from group and individual sessions were reviewed by one of us (M.K.B.) to ensure fidelity and competence. During the remainder of the trial, fidelity was monitored weekly to ensure that content and messages were delivered as intended. As detailed elsewhere,^30^ tailored feedback messages were based on group-specific templates and edited by coaches to enhance personalization, then reviewed by a supervisor to ensure competence and avoid contamination. Monitoring indicated that the treatments were delivered as intended.

### Assessments

Participants completed assessments at baseline, 3 months, and 6 months after a 12-hour fast (nothing except water). Assessments were conducted in the principal investigator’s research laboratory on the extended Virginia Commonwealth University Health campus by trained and certified staff members masked to treatment assignment. Measures were taken in serial and the mean was used in analyses. Participants received $50 for completing follow-up visits plus $5 for transportation costs.

#### Sociodemographic Characteristics

Participants self-reported racial and ethnic identity using NIH categories; an “Another” category with free response option was also provided for participants whose racial or ethnic identity did not align with the NIH categories. Participants self-reported sex assigned at birth, gender identity, work and school status, income, and financial strain. Sociodemographic characteristics were assessed to characterize the sample generalizability and for exploratory moderator analyses.

#### Weight, Height, and BMI

Weight was measured on a calibrated clinic scale in light clothes without shoes. Height was assessed via wall-mounted stadiometer. Body mass index was calculated as weight in kilograms divided by height in meters squared.

#### Waist Circumference

Waist circumference was measured at the midpoint between the highest point of the iliac crest and the lowest part of the costal margin in the midaxillary line using a Gulick tape measure (Sammons Preston).

#### Body Composition

Body composition was assessed via bioelectrical impedance analysis using the BC-418 Segmental Body Composition Analyzer (Tanita). Percentage body fat was the prespecified outcome of interest.

#### Blood Pressure

Blood pressure was measured using a Carescape V100 vital signs monitor (General Electric Co). Cuff size was determined by arm circumference. Participants were seated with feet flat on the floor and forearm supported on a table. After a 5-minute rest, 3 measures were taken from the right arm with 30 seconds or more in between measures.

### Statistical Analysis

Analyses were performed using SAS, version 9.4 (SAS Institute Inc). Mean (SD) values were calculated for continuous variables; frequencies and percentages were calculated for categorical variables. The primary outcome of interest was 6-month weight change (in kilograms). Primary and secondary analyses adhered to the intention-to-treat principle—all participants were analyzed in their assigned treatment groups regardless of adherence, compliance, or study completion. Missing data for all physical outcomes were accounted for using multiple imputation by fully conditional specification in which we generated 25 imputed data sets. Sensitivity analyses were conducted using maximum likelihood estimation and completers; findings were consistent. The primary analysis was a longitudinal (3- and 6-month) generalized linear mixed model analysis of covariance with baseline weight as the covariate, with primary comparisons between each treatment group at 6 months. Comparisons across 3-month outcomes and time were also conducted. Secondary analyses for continuous measures used the same approach, with the baseline covariate changed as appropriate. No other covariates were prespecified. To examine the categorical outcome of a weight loss of 5% or more, we used fully conditional specification multiple imputation in combination with the longitudinal generalized estimating equation approach and compared outcomes between each group. An adjusted α was prespecified given planned comparisons between all 3 groups (α = .0167).

## Results

### Participant Characteristics

Among the 382 participants (mean [SD] age, 21.9 [2.2] years), 316 (82.7%) were female, mean (SD) BMI was 33.5 (4.9), and 222 (58.1%) identified as 1 or more underrepresented race or ethnic groups (Table 1). The sample was diverse with respect to work and school status, and most participants (203 [53.1%]) were single, with a mean (SD) annual income of $21 450 ($15 631). Retention at the primary end point was 83.8% (320 of 382) with no significant differences by race (underrepresented race and ethnicity, 84.7% [188 of 222] vs non-Hispanic White, 82.5% [132 of 160]; *P* = .57), ethnicity (Hispanic or Latinx, 83.7% [41 of 49] vs non-Hispanic, 83.8% [279 of 333]; *P* = .98), sex (male, 81.8% [54 of 66] vs female, 84.2% [266 of 316]; *P* = .64), or treatment group (aBWL, 80.8% [101 of 125], aBWL + BE, 81.4% [105 of 129], and aBWL + SDT, 89.1% [114 of 128]; *P* = .14).

**Table 1.** Characteristics of the REACH Trial Participants at Enrollment

Variable	Participants, No. (%)			
	Overall (N=382)	aBWL group (n=125)	aBWL + BE group (n=129)	aBWL + SDT group (n=128)
Sex assigned at birth^a^				
Female	316 (82.7)	104 (83.2)	105 (81.4)	107 (83.6)
Male	66 (17.3)	21 (16.8)	24 (18.6)	21 (16.4)
Age, mean (SD), y	21.9 (2.2)	21.7 (2.3)	22.0 (2.1)	21.9 (2.1)
BMI, mean (SD)	33.5 (4.9)	33.2 (5.0)	33.9 (4.7)	33.4 (5.0)
Weight, mean (SD), kg	93.1 (17.1)	92.0 (16.8)	94.4 (17.8)	92.9 (16.6)
Ethnicity				
Hispanic or Latinx	49 (12.8)	12 (9.6)	19 (14.7)	18 (14.1)
Non-Hispanic	333 (87.2)	113 (90.4)	110 (85.3)	110 (85.9)
Race				
American Indian or Alaska Native only	3 (0.8)	0	1 (0.8)	2 (1.6)
Asian only	22 (5.8)	7 (5.6)	8 (6.2)	7 (5.5)
Black only	129 (33.8)	49 (39.2)	44 (34.1)	36 (28.1)
Multiracial	30 (7.9)	6 (4.8)	11 (8.5)	13 (10.2)
White only	180 (47.1)	59 (47.2)	59 (45.7)	62 (48.4)
Another race not listed^b^	17 (4.5)	3 (2.4)	6 (4.7)	8 (6.3)
Missing	1 (0.3)	1 (0.8)	0	0
Educational level				
High school (10-12 y)	35 (9.2)	9 (7.2)	10 (7.8)	16 (12.5)
Vocational training	4 (1.0)	0	2 (1.6)	2 (1.6)
Some college (<4 y)	184 (48.2)	63 (50.4)	64 (49.6)	57 (44.5)
College degree	134 (35.1)	43 (34.4)	47 (36.4)	44 (34.4)
Graduate or professional degree	25 (6.5)	10 (8.0)	6 (4.7)	9 (7.0)
Marital status				
Married	20 (5.2)	7 (5.6)	6 (4.7)	7 (5.5)
Single	203 (53.1)	70 (56.0)	67 (51.9)	66 (51.6)
Committed relationship				
Living together	58 (15.2)	12 (9.6)	17 (13.2)	29 (22.7)
Not living together	97 (25.4)	35 (28.0)	39 (30.2)	23 (18.0)
Other	3 (0.8)	1 (0.8)	0	2 (1.6)
Missing	1 (0.3)	0	0	1 (0.8)
Work or school status				
Neither work nor school	8 (2.1)	3 (2.4)	2 (1.6)	3 (2.3)
Work only	118 (30.9)	34 (27.2)	39 (30.2)	45 (35.2)
School only	86 (22.5)	37 (29.6)	21 (16.3)	28 (21.9)
School and work	170 (44.5)	51 (40.8)	67 (51.9)	52 (40.6)
Income, mean (SD), $	21 450 (15 631)	20 517 (14 275)	22 446 (15 874)	21 358 (16 684)
Difficulty living on total household income				
Not at all	118 (31.0)	42 (33.9)	35 (27.1)	41 (32.0)
Somewhat	199 (52.2)	63 (50.8)	67 (51.9)	69 (53.9)
Very	46 (12.1)	11 (8.9)	20 (15.5)	15 (11.7)
Extremely	18 (4.7)	8 (6.5)	7 (5.4)	3 (2.3)

Abbreviations: aBWL, adapted behavioral weight loss; aBWL + BE, adapted behavioral weight loss plus behavioral economics; aBWL + SDT, adapted behavioral weight loss plus self-determination theory; BMI, body mass index (calculated as weight in kilograms divided by height in meters squared); REACH, Richmond Emerging Adults Choosing Health.

^a^ Sex assigned at birth and gender identity were both assessed and correspondence was 100%.

^b^ Included individuals who wrote in their race because none of the listed National Institutes of Health categories aligned with their identity; some participants identified as Latino or Hispanic, and some individuals identified as Middle Eastern.

### Change in Weight and Cardiometabolic Risk Factors

**Figure 2.**
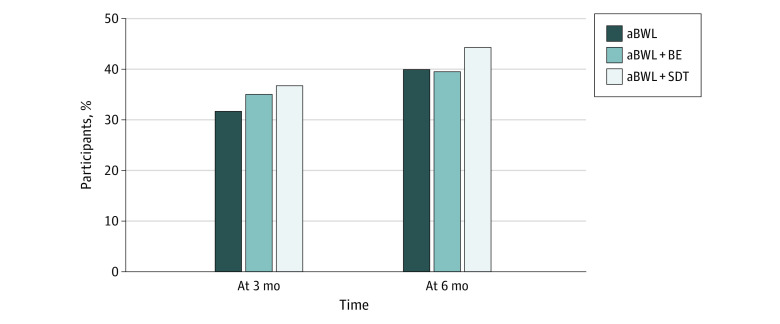
Proportion of Participants Achieving a Weight Loss of 5% or More by Intervention Group aBWL indicates developmentally adapted behavioral weight loss; BE, behavioral economics; and SDT, self-determination theory.

Table 2 depicts change in primary and secondary outcomes. The prespecified primary outcome, mean (SE) weight change at 6 months, was −3.22 (0.55) kg in the aBWL group, −3.47 (0.55) kg in the aBWL + BE group, and −3.40 (0.53) kg in the aBWL + SDT group. The reduction in weight over time was statistically significant in all groups (all *P* < .001), but there were no observed differences between groups (aBWL vs aBWL + BE: difference, −0.25 kg [95% CI, −1.79 to 1.29 kg]; *P* = .75; aBWL vs aBWL + SDT: difference, −0.18 kg [95% CI, −1.67 to 1.31 kg]; *P* = .81; and aBWL + SDT vs aBWL + BE: difference, 0.07 kg [95% CI, −1.45 to 1.59 kg]; *P* = .93). Parallel findings were observed for percentage weight change and change in BMI, such that statistically significant improvements were observed over time, with no between-group differences. The proportions of participants within each group who achieved a weight loss of 5% or more at the primary end point of 6 months were 40.0% in the aBWL group (50 of 125), 39.8% in the aBWL + BE group (51 of 128), and 44.2% in the aBWL + SDT group (57 of 129) (Figure 2), which were not statistically different across groups (aBWL vs aBWL + BE, *P* = .89; aBWL vs aBWL + SDT, *P* = .45; aBWL + SDT vs aBWL + BE, *P* = .54). Statistically significant improvements were also observed in waist circumference, percentage body fat, and systolic blood pressure, with no significant between-group differences (Table 2). The aBWL + BE group was the only group to experience statistically significant mean (SD) improvements in diastolic blood pressure at 6 months (−1.60 [+0.56] mm Hg; *P* = .001), but differences were not significantly different from the aBWL group ( +0.13 [+0.58] mm Hg; *P* = .049) or the aBWL + SDT group (−0.42 [0.52] mm Hg; *P* = .24). No adverse events were determined to be trial related.

**Table 2.** Summary of Primary and Secondary Results

Outcome^a^	aBWL group	aBWL + BE group	aBWL + SDT group	Pairwise comparisons					
				aBWL group vs aBWL + BE group		aBWL group vs aBWL + SDT group		aBWL + SDT group vs aBWL + BE group	
				Difference (95% CI)	*P* value	Difference (95% CI)	*P* value	Difference (95% CI)	*P* value
Weight change, mean (SE), kg	NA	NA	NA	−0.21 (−1.44 to 1.03)	.74	−0.13 (−1.33 to 1.08)	.84	0.08 (−1.13 to 1.30)	.89
At 3 mo	−2.78 (0.37)	−2.95 (0.36)	−2.86 (0.35)	−0.17 (−1.18 to 0.85)	.75	−0.07 (−1.08 to 0.94)	.89	0.10 (−0.89 to 1.08)	.85
At 6 mo	−3.22 (0.55)	−3.47 (0.55)	−3.40 (0.53)	−0.25 (−1.79 to 1.29)	.75	−0.18 (−1.67 to 1.31)	.81	0.07 (−1.45 to 1.59)	.93
% Weight change	NA	NA	NA	−0.28 (−1.62 to 1.05)	.68	−0.17 (−1.47 to 1.14)	.80	0.12 (−1.20 to 1.43)	.86
At 3 mo	−3.06 (0.40)	−3.25 (0.39)	−3.14 (0.38)	−0.20 (−1.29 to 0.90)	.72	−0.08 (−1.17 to 1.00)	.88	0.11 (−0.95 to 1.17)	.84
At 6 mo	−3.51 (0.60)	−3.88 (0.60)	−3.76 (0.58)	−0.37 (−2.03 to 1.29)	.66	−0.25 (−1.86 to 1.37)	.76	0.12 (−1.53 to 1.77)	.89
Change in BMI	NA	NA	NA	−0.14 (−0.56 to 0.29)	.53	−0.13 (−0.56 to 0.29)	.54	0.01 (−0.42 to 0.43)	.97
At 3 mo	−1.07 (0.13)	−1.19 (0.13)	−1.17 (0.12)	−0.12 (−0.48 to 0.24)	.51	−0.10 (−0.45 to 0.26)	.59	0.02 (−0.32 to 0.37)	.90
At 6 mo	−1.27 (0.19)	−1.43 (0.19)	−1.44 (0.19)	−0.16 (−0.70 to 0.38)	.56	−0.17 (−0.69 to 0.36)	.53	−0.01 (−0.54 to 0.53)	.98
Change in waist circumference, cm	NA	NA	NA	−0.42 (−1.72 to 0.89)	.53	−0.38 (−1.67 to 0.92)	.57	0.04 (−1.23 to 1.30)	.95
At 3 mo	−2.85 (0.42)	−3.54 (0.40)	−3.28 (0.40)	−0.69 (−1.83 to 0.46)	.24	−0.43 (−1.56 to 0.70)	.45	0.26 (−0.84 to 1.36)	.64
At 6 mo	−4.56 (0.58)	−4.70 (0.58)	−4.89 (0.57)	−0.14 (−1.76 to 1.48)	.86	−0.33 (−1.94 to 1.29)	.69	−0.18 (−1.77 to 1.40)	.82
Body fat percentage	NA	NA	NA	−0.30 (−0.97 to 0.38)	.39	−0.37 (−1.04 to 0.29)	.27	−0.08 (−0.72 to 0.57)	.81
At 3 mo	−1.20 (0.22)	−1.47 (0.22)	−1.58 (0.22)	−0.27 (−0.89 to 0.36)	.40	−0.38 (−0.98 to 0.23)	.22	−0.11 (−0.71 to 0.49)	.72
At 6 mo	−1.52 (0.30)	−1.84 (0.28)	−1.89 (0.28)	−0.32 (−1.13 to 0.48)	.43	−0.37 (−1.17 to 0.42)	.36	−0.05 (−0.81 to 0.72)	.91
Systolic blood pressure, mm Hg	NA	NA	NA	−1.09 (−2.73 to 0.55)	.19	−1.32 (−2.90 to 0.26)	.10	−0.23 (−1.79 to 1.33)	.77
At 3 mo	−2.27 (0.67)	−2.71 (0.64)	−3.34 (0.63)	−0.44 (−2.28 to 1.40)	.64	−1.06 (−2.87 to 0.75)	.25	−0.62 (−2.36 to 1.12)	.48
At 6 mo	−1.12 (0.73)	−2.86 (0.71)	−2.70 (0.66)	−1.74 (−3.74 to 0.26)	.09	−1.58 (−3.49 to 0.33)	.11	0.16 (−1.73 to 2.04)	.87
Diastolic blood pressure, mm Hg	NA	NA	NA	−1.30 (−2.59 to −0.01)	.049	−0.57 (−1.82 to 0.67)	.37	0.71 (−0.50 to 1.95)	.24
At 3 mo	−0.22 (0.55)	−1.09 (0.52)	−0.81 (0.51)	−0.87 (−2.37 to 0.63)	.25	−0.60 (−2.08 to 0.88)	.43	0.27 (−1.14 to 1.69)	.70
At 6 mo	0.13 (0.58)	−1.60 (0.56)	−0.42 (0.52)	−1.73 (−3.31 to −0.14)	.03	−0.55 (−2.06 to 0.96)	.47	1.18 (−0.31 to 2.66)	.12

Abbreviations: aBWL, adapted behavioral weight loss; aBWL + BE, adapted behavioral weight loss plus behavioral economics; aBWL + SDT, adapted behavioral weight loss plus self-determination theory; BMI, body mass index (calculated as weight in kilograms divided by height in meters squared); NA, not applicable.

^a^ Changes over time are all statistically significant (*P* < .001) for all outcomes except diastolic blood pressure; in addition, changes in systolic blood pressure in the aBWL group were *P* = .001 and *P* = .13 at 3 and 6 months, respectively.

### Self-monitoring by Treatment Group

The mean (SD) total number of days of self-weighing during the 6-month period was 68.5 (4.3) days in the aBWL group, 84.9 (4.4) days in the aBWL + BE group, and 72.1 (4.3) days in the aBWL + SDT group (aBWL group vs aBWL + BE group, *P* = .02; aBWL group vs aBWL + SDT group, *P* = .82; and aBWL + SDT group vs aBWL + BE group, *P* = .10). The mean (SD) number of dietary self-monitoring days during the 6-month period was 65.8 (4.1) days in the aBWL group, 71.6 (4.2) days in the aBWL + BE group, and 57.9 (4.1) days in the aBWL + SDT group (aBWL group vs aBWL + BE group, *P* = .60; aBWL group vs aBWL + SDT group, *P* = .36; and aBWL + SDT group vs aBWL + BE group, *P* = .06).

## Discussion

To our knowledge, the REACH trial was the first large-scale randomized clinical trial for weight management designed specifically for emerging adults aged 18 to 25 years. Results suggest that all tested interventions resulted in clinically significant^1^ weight loss. Contrary to our hypotheses, neither of the motivational enhancements were associated with greater weight loss than the adapted standard intervention. This lack of observed effect may be owing to the strength of the developmentally adapted program that served as the core for all 3 groups. It is also plausible that neither of the motivational enhancements tested were strong enough to result in an additive effect, despite the previously demonstrated promise of these paradigms.^15,22,23^ Outcomes in all groups—including the adapted standard group—compare favorably with outcomes in previous trials among young adults up to 35 years of age,^10,11,12,13,14,17^ including majority digital interventions among young adults,^10,11,12,13,14^ as well the personal coaching group in the CITY (Cell Phone Intervention for You) trial^12^ that included 6 weekly group sessions and monthly coaching calls.

A recent meta-analysis of weight loss interventions among young adults (age, 18-35 years) reported nonsignificant reductions in weight of −1.2 kg with interventions longer than 3 months,^13^ whereas the interventions in the REACH trial produced weight losses nearly 3 times that amount. Similarly modest reductions in waist circumference of less than 1 cm were reported in the meta-analysis,^13^ whereas all groups in the REACH trial had reductions greater than 4.5 cm, which is clinically meaningful given the cardiometabolic risks associated with abdominal adiposity.^1^ All of our interventions included adaptations grounded in formative work with the population,^8^ behavior change techniques recommended for e-Health weight loss interventions for young adults,^14^ and all key components recommended for technology-based weight loss.^34^ These components likely contributed to the clinically meaningful effects observed for all groups with a primarily digital program—at the same time, the evidence-based behavior change techniques and developmental adaptations included may have contributed to the null effects as the motivational enhancements were insufficient to promote an additive effect beyond the core adapted program.

Another factor that may have contributed to the lack of differences between groups was the heterogeneity in treatment response. Variability in weight loss treatment response is a challenge,^35,36,37,38^ but the magnitude of variability observed in this sample of emerging adults was even larger than anticipated.^30^ A previous study among young adults found that a subgroup of participants gained 10% or more of their body weight even while enrolled in a weight gain prevention trial.^38^ There might be a subgroup of emerging adults who require a more intensive program than offered by any of the current interventions. It is also possible that participant characteristics could influence response to these motivational enhancements. In future work it will be critical to explore moderators of treatment response to advance our understanding of which intervention works best for specific individuals based on sociodemographic and/or psychosocial characteristics to assist with treatment matching efforts for emerging adults.

### Limitations and Strengths

This study has some limitations. First, enrollment of young men was low. This is a well-established challenge in the field,^39^ and concerns are lessened given evidence that men lose more weight than women within BWL trials.^40^ Nevertheless, additional work is needed to enhance recruitment of young men within lifestyle intervention trials, and gender identity–specific programming might be an important consideration. In addition, this trial was conducted at a single site within the southeast region of the US—thus, findings may not be generalizable to emerging adults in other geographic regions or other countries. Furthermore, the 6-month primary end point is relatively short term, particularly given the challenge of weight loss maintenance.^41,42,43^ However, this duration corresponded to the end of treatment, was double the length of our previous pilot trials with emerging adults,^15,16^ and the intervention duration is comparable to recent reports.^13^

This study also has several strengths. This was the first large-scale trial focused specifically on student and nonstudent emerging adults, to our knowledge. We also tested interventions grounded in theory and formative work that included evidence-based behavior change techniques. Furthermore, we used a randomized design with strong internal validity wherein we isolated the effect of the motivational enhancements beyond that of the core program. We demonstrated strong retention, with appropriate handling of missing data, and physical measures were objectively assessed by masked assessors following a standardized protocol. Finally, we enrolled a diverse sample of emerging adults with respect to race and ethnicity, work and school status, and socioeconomic status, which enhances the generalizability of our findings.

## Conclusions

Results of this randomized clinical trial indicate that clinically significant improvements in weight and other cardiometabolic risk factors can be achieved among 18- to 25-year-old emerging adults via a primarily digital intervention, but additional data are needed on long-term effects. Neither of the motivational enhancements were sufficient to promote greater reductions in adiposity compared with our developmentally adapted standard intervention. Continued efforts are needed to optimize lifestyle interventions for individuals in this high-risk developmental period and to determine which intervention works best for specific individuals based on sociodemographic and/or psychosocial characteristics.

## Notes

Supplement 1.
Trial Protocol

Supplement 2.
Data Sharing Statement
