# Supplementary material for: Effect of a Lifestyle Intervention on Cardiometabolic Health Among Emerging Adults: A Randomized Clinical Trial
Source: JAMA Netw Open. 2022 Sep 19;5(9):e2231903. doi: 10.1001/jamanetworkopen.2022.31903 (PMC9486452; doi:10.1001/jamanetworkopen.2022.31903)
Supplement: Supplement 1. — Trial Protocol [file jamanetwopen-e2231903-s001.pdf]

REACH Trial Protocol
(*Richmond Emerging Adults Choosing Health*)
Version 1 – 09/2015
Version 2 – 01/2017
Version 3 – 08/2018

*Note: *Version changes reflected changes to ancillary questionnaires only; no changes were made to the design and methods, primary and secondary outcome measures, or statistical analysis plan from the original protocol version.*

1. Executive Summary

Over 40% of emerging adults ages 18-25 meet criteria for overweight or obesity, with rates exceeding 50% in African American and Hispanic / Latino populations [1]. Further, obesity during these years is associated with increased risk of diabetes, hypertension and hyperlipidemia [2]. Despite being at substantial risk, emerging adults are markedly underrepresented in adult behavioral weight loss (BWL) programs, representing less than 1% of participants enrolled in NIH-funded adult BWL trials [3], and recruitment of this population has been noted as a challenge [3-5]. Further, data indicate that the small number of 18-25 year olds who do enroll in standard programs do not fare as well as their adult counterparts, as evidenced by poorer engagement, retention and weight losses [3,6]. In fact, this age group in particular fares worse even relative to young adults (i.e., up to 35 years), with some studies noting retention of 18-25 year olds to be as low as 25-33% within the context of adult weight loss programs [3,6]. Evidence-based BWL programs exist for pediatric [7], adolescent [8] and adult [9] populations, but an evidence-based gold standard does not exist for emerging adults despite data suggesting that extant adult programs are not meeting their needs. Although this is increasingly recognized as a key time for intervention, limited work has been done in the area to date. To our knowledge, other than our own pilot work, no programs have specifically targeted emerging adults 18-25 years for weight loss. Lack of effective treatment in this population could have a profound impact on the individual and public health level; thus, novel and effective approaches are urgently needed.

The Richmond Emerging Adults Choosing Health (REACH) Trial is a 3-arm randomized controlled trial designed to test the relative efficacy of two novel motivational approaches to weight loss with 18-25 year olds and an adapted version of gold standard BWL. Participants will be 381 18-25 year olds with a BMI of 25-45 kg/m^2 who will be randomized to 1 of 3 arms: 1) Adapted Behavioral Weight Loss (BWL), 2) BWL + Autonomous Motivation (BWL+Aut) or 3) BWL + Extrinsic Motivation (BWL+Ext). All participants who are enrolled into treatment will receive the same evidence-based BWL program that has been tailored to emphasize problem areas of specific relevance to this age group. Lessons include weekly videos and PDFs, as well as supporting materials and resources; this content was developed for our previous work and has been shown to produce significant weight losses in emerging adults [10-12]. Participants will be given personalized weight loss, dietary and physical activity goals, and guidelines for self-monitoring of diet and weight. All participants will receive training in core BWL strategies and techniques to help them achieve these goals, and will receive personalized feedback on their progress throughout the program. The key differences between the arms are the explicit emphasis on facilitating autonomy, competence and relatedness and a Self-Determination Theory [13-15] framework in the BWL+Aut arm and the use of extrinsic monetary rewards and a behavioral economics [16-17] framework in the BWL+Ext arm.

The primary aim of the trial is to test the relative efficacy of the three arms on percent weight change at 6 months with a priori hypotheses that the BWL+Aut and the BWL+Ext conditions will produce greater weight loss outcomes at 6 months, compared with BWL. We further hypothesize that BWL+Ext will achieve greater weight loss at 6 months, relative to BWL+Aut. Secondary aims are to evaluate the relative efficacy of the three arms on secondary outcomes of interest (e.g., blood pressure, waist circumference, body composition, physical activity) at 6 months. REACH will also explore whether there are differential rates of weight maintenance across all arms from 6 to 12 months, and, if hypotheses for the primary aim are supported, we will examine mediators of treatment outcomes consistent with our theoretical framework (e.g., autonomous motivation, extrinsic motivation).

2. Background

Obesity is a major medical and public health threat and emerging adults are at particularly high risk. In the United States, obesity is the second leading cause of preventable death [18] and is associated with $147 billion in medical costs annually [19]. The period between 18 and 25 years of age, known as "emerging adulthood," [20-21] is a particularly high-risk period [22]. Over 40% of emerging adults ages 18-25 meet criteria for overweight or obesity, with rates exceeding 50% in African-American and Hispanic / Latino populations [1]. Further, obesity during these years is associated with increased risk of diabetes, hypertension and hyperlipidemia [2]. Extant data also indicate this transition is associated with a host of unhealthy weight related behaviors, including sharp declines in physical activity [23], increased sedentary behaviors [24], increased consumption of fast food [25-26] and sugar sweetened beverages [27]. Additionally, documented high rates of perceived stress [28], depression [29], and substance abuse [1] during emerging adulthood signal that this is perhaps a critical time for intervention to promote healthy lifestyle behaviors.

Although there are empirically supported lifestyle interventions targeting children [7] and adolescents [8], individuals over age 18 are typically considered "adults" and included in adult behavioral weight loss (BWL) programs. Adult BWL programs consistently produce weight losses of 8-10kg in the initial 6 months of treatment [9,30], and weight losses of this magnitude have been shown to reduce risk for diabetes and cardiovascular disease [31-33]. However, there is evidence that these programs are not meeting the needs of emerging adults. A secondary analysis of several large, NIH-funded BWL trials revealed young adults (18-35 years) were markedly underrepresented – of note, results were most striking for emerging adults 18-25 years of age, who represented less than 1% of participants [3]. Moreover, data indicate that this age group (18-25 years) fares worse relative to older adults and even compared with young adults (i.e., up to 35 years) in terms of program engagement and weight losses [3,6]. These data are not surprising given the developmental considerations and substantial life transitions unique to emerging adulthood. In fact, researchers have argued that it makes little sense to lump together individuals in their late teens and early twenties with those in their late twenties and early thirties, given the dynamic quality of the transition into adulthood and the considerable distinctions between the late teens / early twenties and the thirties in terms of education, work environment, marriage and family status [20-22].

Although this is increasingly recognized as a key time for intervention, limited work has been done in the area to date. Several pilot studies have targeted college students for weight gain prevention [34-36] or weight loss [37-38] with promising initial short-term outcomes. Additionally, NIH funded a consortium of ongoing studies (EARLY Trials) focused on weight control during young adulthood more broadly (18-35 years) [39]. However, none of these trials focused on weight loss specifically for 18-25 year olds and limited their sample to this age range, and many of these trials focused on weight gain prevention as opposed to weight loss. Moreover, other than our own recent pilot work [38], to our knowledge, behavioral weight loss programs specifically targeting emerging adults 18-25 years of age are lacking [5].

REACH is the first fully powered trial to target 18-25 year olds for weight loss. We conducted extensive formative and pilot work with 18-25 year olds with overweight or obesity to inform the current study [40]. Data supported the need for a reduced intensity program, relative to standard adult programs. Further, findings reflected a desire for individual-level treatment that relies primarily on technology-based contact. Of particular note, lack of motivation was identified as a central barrier to weight management, and findings indicate that motivation needs to be addressed in the program structure and content in order to maximize appeal and engagement with this age group. Of note, motivation is linked to better outcomes within weight control trials [41-42]. However, while many BWL programs assess motivation, standard adult programs do not target motivation explicitly and do not address different types of motivation in program design or content. Given the documented challenges engaging emerging adults in adult BWL programs, and the fact that motivation was identified as a key barrier to weight loss, finding novel ways to enhance motivation may be essential to improving engagement and weight loss outcomes with this population, particularly within the context of a reduced touch, technology-mediated intervention.

REACH is delivered primarily via an interactive technology platform and is designed to enhance motivation and program engagement to improve weight loss, cardiometabolic and psychological outcomes with this population. Efforts to enhance motivation in this population can be approached in different ways – our formative and pilot data suggest that two key drivers of motivation among 18-25 year olds are autonomy and money [40]. Thus, two

potentially viable options to facilitate motivation with a BWL program designed for this age group are: 1) to emphasize the importance of autonomous self-regulation and intrinsic motivation for health behavior change, or 2) to foster extrinsic motivation through the use of external reward contingencies. Both approaches may have the potential to improve adherence to behavioral recommendations and weight loss (see Figure). REACH will test these two approaches head to head.

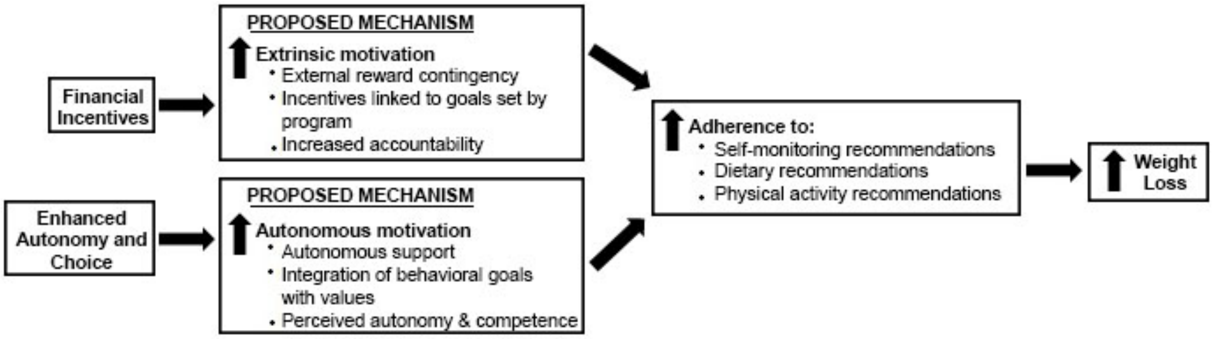

A reduced intensity, enhanced motivation intervention may be a novel yet pragmatic approach to weight loss with this population. Moreover, a head-to-head comparison of programs designed to facilitate either extrinsic or autonomous motivation is of both theoretical and practical significance. The use of rewards to encourage behavior change is central to learning theory and a core tenet of BWL [9,30]. Previous studies have provided token reinforcers to promote adherence and behavior change in weight control trials [10-11,43-44]. Research has also demonstrated that programs offering financial incentives yield superior initial weight losses compared to programs without financial rewards [16,45-47]. Further, our formative work indicate that money may be a particularly powerful method of facilitating motivation and engagement with 18-25 year olds. Thus, providing small financial incentives for meeting behavioral and weight loss targets might be a practical sustainable method of promoting weight loss in a reduced-intensity program targeting 18-25 year olds. Such an approach also has considerable appeal from a policy perspective because of its potential for wide scale implementation; indeed, financial incentives are being used by over 67% of large employers to motivate health behavior change and reduce healthcare costs [48]. Moreover, preliminary data suggest offering small financial incentives for self-monitoring and weight loss produces clinically significant weight loss with this age group. However, once monetary incentives are removed, substantial rates of weight regain are often observed [16,45-46], and it is unknown whether incentives may produce sustained weight loss in this age group.

In contrast, autonomous self-regulation is believed to be particularly important for sustained behavior change [13-15,41-42]. Research has demonstrated a positive relationship between autonomous motivation and health behavior change in a variety of domains, including weight loss; and data indicate perceived autonomy support predicts better long-term weight loss than directive support [41-42,49]. Additionally, autonomy and volition were identified as critically important features of a weight loss program for this age group and a desire for increased autonomy is characteristic of the developmental transition from adolescence into early adulthood [20-21]. However, adult BWL programs are typically directive in nature and not designed to promote autonomy. Further, the few programs that have targeted autonomous motivation explicitly have been quite intensive [50-51], which is not consistent with the needs and preferences of this age group and holds less potential for dissemination. According to Self-Determination Theory [13-15], climates that provide choice and opportunity for self-direction are thought to enhance intrinsic motivation by promoting autonomy, and goals that are of personal importance and consistent with one's values are more autonomous. As such, incorporating opportunities for choice and highlighting autonomy around behavioral goals might be effective methods for promoting autonomy within a reduced-intensity program delivered primarily via a technology platform. Moreover, data from our pilot work indicate that such an approach is feasible and yields clinically significant weight losses with 18-25 year olds.

<u>Summary of Potential Impact</u>: REACH is the first fully powered trial to target a high-risk emerging adult population for whom no standard treatment exists. Given the substantial proportion of 18-25 year olds who already meet criteria for overweight or obesity and the looming cardiometabolic risks associated with weight gain and obesity during these years, intervention in this population is critical. Thus, the current study is clinically significant. Furthermore, the current trial stands to have an important scientific impact as it will be the first trial to directly

compare a health behavior intervention grounded in Self-Determination Theory to one rooted in behavioral economics that uses financial incentives. Not only will these two distinct theoretical approaches be tested head-to-head to determine which produces better engagement and weight loss initially, but the no-treatment contact follow up period will allow for an examination of the differential patterns of maintenance and / or erosion of behaviors over time once the intervention is removed. Finally, if clinically significant weight losses are achieved through any or all of these reduced intensity programs, which are delivered primarily via a technology platform, findings could inform a scalable model for treatment to meet the needs of this unique population.

3. Overview of Trial Design

3.1 Trial Design

REACH is a 3-armed randomized controlled trial, comparing the relative efficacy of three adapted behavioral weight loss programs delivered using minimal in-person treatment contact augmented with an interactive technology platform. Group 1 represents the closest approximation to the gold standard program; Group 2 is grounded in Self-Determination theory and emphasizes autonomy and intrinsic motivation to enhance engagement and weight loss; Group 3 is rooted in behavioral economics and incorporates modest financial incentives as extrinsic rewards to promote adherence behaviors and weight loss. The trial will enroll 381 emerging adults in the Richmond Metropolitan Area, age 18-25 years with a BMI of 25-45 kg/m^2. These participants will be randomly assigned to 1 of 3 groups:

1) Adapted BWL (BWL), (N=127)
2) BWL + Autonomous Motivation (BWL+Aut), (N=127)
3) BWL + Extrinsic Motivation (BWL+Ext), (N=127)

All arms will receive a 6-month treatment program, consisting of 1 in-person group session, 1 in-person individual session, followed by weekly intervention contact via an interactive technology platform through 6 months. All participants will continue to be followed for a no-treatment follow-up period through 12 months.

3.2 Specific Aims and Hypotheses

Primary Aim: To test the relative efficacy of BWL, BWL+Aut and BWL+Ext on weight change at 6 months.

Secondary Aim: To evaluate the relative efficacy of BWL, BWL+Aut and BWL+Ext on secondary outcomes of interest (e.g., blood pressure, waist circumference, body composition, physical activity) at 6 months.

Exploratory Aim 1: Given evidence that once financial incentives are removed, substantial regain occurs [16,45-46], and that autonomous self-regulation is associated with better longer-term outcomes [41-42], we will explore whether there are differential rates of weight maintenance across all arms from 6 to 12 months.

Exploratory Aim 2: If hypotheses for the primary aim are supported, we will examine mediators of outcomes consistent with our theoretical framework (e.g., autonomous motivation, extrinsic motivation).

3.3 Sample Size Justification

Using estimates from our pilot data, we expect a standard deviation (SD) of 5.0 in each of the three arms with respect to the planned comparisons between arms. This is equivalent to a medium effect size (d=.5) based on guidelines suggested by Cohen [52]. Adjusting for 3 planned group comparisons, and allowing for a 15% attrition rate at 6 months, a sample size of 127 per arm (total study N=381) would allow us to detect a clinically meaningful difference of 2.5 kg with 90% power at the adjusted α=.0167 level of significance. An important exploratory aim is to compare the maintenance of weight loss from 6 months to 12 months. Given that our estimates of SD are based on studies of shorter duration, we examined a series of scenarios to determine our available statistical power using a variety of SDs, while maintaining the same threshold for clinical significance of 2.5 kg. If the SD were to increase to 5.5 at 12 months (d=.45) and attrition were not to exceed 15%, we would still have 82% power for 3 comparisons conducted at the adjusted level of α=.0167. Power drops slightly to 76% when the SD

rises to 6.0 (d=.42) for 3 comparisons conducted at the adjusted level of α=.0167. For any comparison conducted at the nominal alpha = .05 level, we retain 80% power up to a SD=6.5 (d=.38).

4. Study Population

REACH seeks to recruit 381 men and women who are 18-25 years old, with a BMI of 25 to 45 kg/m^2, who respond to recruitment ads, meet standard medical / safety inclusion criteria for a BWL trial (e.g., no uncontrolled medical conditions, not pregnant or lactating), who have not lost >5% of their body weight within the last 3 months, complete all screening appointments, and are willing to be randomized to one of three arms, with $\geq$30% of participants being male and $\geq$40% self-identifying as belonging to an NIH-identified racial / ethnic minority group.

*Individuals will be considered **eligible** if they meet the following criteria:*

1) Age between 18 and 25 years. This is a unique developmental period – large numbers of 18-25 year-olds meet criteria for overweight or obesity and significant weight gain occurs during these years, yet this group is all but non-existent in adult BWL studies; and data suggest they do not respond as well to standard adult programs. Thus, this study seeks to specifically recruit men and women in this high-risk age group.
2) Body mass index (BMI) between 25 and 45 kg/m^2. Based upon the Guidelines for Management of Overweight and Obesity in Adults (AHA/ACC/TOS, 2013), weight loss is recommended for individuals with a BMI $\geq$ 25. Individuals with a BMI >45 have more medical co-morbidities, and may be less ideal candidates for unsupervised exercise and a low intensity program such as the one proposed in this application. Thus, anyone with a BMI less than 25 or greater than 45 will be considered ineligible.
3) Men and women will be recruited for this study, and the study goal will be to recruit at least 30% men.
4) All ethnic groups will be recruited for this study, with a study goal of recruiting at least 40% of participants from NIH-identified racial / ethnic minority groups.

*Individuals will be **ineligible** if they report any of the following:*

1) Report an uncontrolled medical condition that may pose a safety issue given the recommendations for diet and unsupervised physical activity in the current study (e.g., uncontrolled hypertension). We will refer individuals who have blood pressure levels >140 / 90 mmHg as part of baseline screening to their physician and exclude them from this study unless their treating physician determines s/he is a suitable candidate based on the specifics of the protocol and provides written medical consent for participation. Participants who report a history of or current treatment for medical conditions (e.g., hypertension, diabetes) will be asked to obtain MD consent to participate.
2) Other health problems or medications which may influence the ability to walk for physical activity or be associated with unintentional weight change.
3) Cancer treatment within the past 5 years.
4) Report a heart condition, chest pain during periods of activity or rest, or loss of consciousness on the Physical Activity Readiness Questionnaire (PAR-Q), which will be administered during prescreen. Individuals endorsing joint problems, prescription medication usage, or other medical conditions that could limit exercise will be required to obtain written physician consent to participate.
5) Report a history of, or current diagnosis of anorexia nervosa or bulimia nervosa, or any current compensatory behaviors (e.g., vomiting, laxative use, excessive exercise).
6) Current symptoms of alcohol or substance dependence.
7) Planning to move from the area within the study period.
8) Currently pregnant, pregnant within the past 6 months, currently breastfeeding or planning to become pregnant within the next 12 months.
9) Hospitalization for depression or other psychiatric disorder within the past 12 months. History of psychotic disorder or bipolar disorder.
10) Participation in another research study that may interfere with this study.
11) Currently participating in a weight loss program and/or taking weight loss medication or lost $\geq$ 5% of body weight during the past 3 months.
12) Failure to complete the screening appointments.

5. Recruitment and Screening

Participants will be recruited in cohorts of approximately 63 (~21 randomized to each arm). Recruitment strategies proven successful in our previous work with emerging adults will be used, including: newspapers (both print and online), television, radio, online radio (e.g., Pandora), billboards, print flyers, bus ads (e.g., RAMRide, GRTC), email blasts (e.g., paid mass mailings and posts to listservs such as the VCU TelegRAM), digital media campaigns, various social media platforms (e.g., Facebook, Twitter, Instagram), and postings on local area Internet forums (e.g., Reddit, Craigslist), community contacts, and site-specific recruitment at locations where the target population likely live and work.

To increase male participation, targeted male recruitment ads will be used (e.g., targeted generic communication – including the word "men" in ads, using pictures of men). In addition, we will recruit specifically in outlets / venues that have high male representation (e.g., local police and fire departments, fraternities, gyms). To increase minority participation, we will build upon our established relationships in the African American (AA) community within the greater Richmond area; we will directly work with local churches, schools and community centers serving high proportions of AA youth and emerging adults. Further, advertisements will be placed in newspapers with high circulation in minority communities and personal contacts will be made and recruitment fliers will be sent to local agencies that serve minority populations (e.g., Black churches, YMCA, Office of Multicultural Student Affairs, Black Student Union).

The use of recruitment websites was recommended in our formative work and has been very well received in our previous studies with this age group. The website address is used in all recruitment materials to allow potential participants to visit and learn more at their convenience. An overview of the study and requirements (e.g., specifics about the treatment groups, eligibility requirements, follow up visits) is provided and interested participants can click through to a secure system to complete an online screening questionnaire to determine preliminary eligibility. Dynamic and appealing recruitment websites and web-based screening has been a critical part of our success in recruiting 18-25 year olds to date. Participants will provide electronic consent prior to completing a screening questionnaire via a secure study screening website. Those individuals who prefer to complete the screening questions by phone as opposed to via the secure website will be given the opportunity to do so; in this case, participants will provide verbal consent prior to answering any screening questions. Participants who are eligible based on initial screening will be invited to attend an orientation session where detailed information about the study purpose and procedures will be provided, and the concept of random assignment will be described. Individuals will be given an opportunity to ask specific questions regarding the study. Those who remain interested will begin the informed consent process, which will include the consent form being read aloud and referencing relevant portions of the earlier presentation using visuals providing an opportunity for any remaining questions to be asked. Then those who wish to participate will review and sign a consent form approved by the Institutional Review Board of Virginia Commonwealth University at the conclusion of the orientation visit. This study is subject to HIPAA regulations as deemed by the VCU IRB. Details pertaining to the use and disclosure of protected health information, per IRB and HIPAA guidelines, is outlined in the participant informed consent form. We adhere to strict procedures and protocol to protect participant data and participants' interests and safety within the proposed trial.

6. Randomization

We will employ a variably-sized permuted block randomization procedure for the assignment of participants into BWL, BWL+Aut or BWL+Ext arms. Randomization will be stratified based on sex, baseline BMI and race/ethnicity to help ensure there are no differential baseline differences across arms on these characteristics to impact the outcomes. The randomization scheme will be developed by Dr. Fava, the trial statistician, with treatment assignments placed in password-protected spreadsheets by cohort for eligible participants and provided to study staff at the time of randomization. Participants who consent and meet eligibility based on a completed baseline assessment will be eligible for randomization. In order for a participant to be randomized, he/she must have signed the informed consent, be in the eligible BMI and age categories, not report any of the health problems described above for ineligibility, have physician permission to join the study if deemed necessary based on health parameters (see eligibility), complete a baseline assessment visit, and report being available for the time/place that the initial treatment session is being conducted.

7. Retention

A systematic protocol will be followed to minimize missed treatment contacts and avoid non-use attrition. We will follow an established protocol that we have successfully implemented in our previous work to maximize engagement and use of the website throughout the trial. The protocol outlines frequency and type of reminders and allows for specific strategies for re-engagement. We have used a similar protocol in our ongoing weight gain prevention trial [43,53] which involves a very low intensity web platform over 3 years, and monthly engagement via the website has averaged 60-70% through 2 years. Similar strategies were also used in our pilot for this proposal.

Attendance at assessment visits is critical. At baseline, we will obtain names and addresses of several friends or family who can be contacted if we are unable to reach a participant, and we will ask for participants' consent to contact his/her physician to obtain weight data if unable to collect it directly. For all visits, participants will receive an email with a link to an online scheduling system, enabling them to select the appointment that best suits their schedule. Once participants schedule, they will receive a confirmation email including a reminder of how to prepare for the visit. Early morning, daytime and weekend appointments will be available to offer maximum flexibility and reduce barriers. Participants will receive a reminder email 2 days before their visit, and a reminder text the day before the visit. Participants will receive $50 for completing each of the 3-, 6-month visits and $75 for the 12-month visit. In addition, participants will receive $5 for completing each of the brief online surveys sent out at 2 weeks, 2 months, and 4 months post treatment launch.

8. Interventions

All participants who are enrolled into treatment will receive the same BWL lesson content that has been tailored to emphasize problem areas of specific relevance to this age group. Participants will receive 1 group-based treatment session and 1 individual treatment session to further personalize goals, followed by an interactive technology platform that includes weekly video and PDF lessons, text prompts and reminders, a self-monitoring platform and personalized feedback on progress. Participants will be given weight loss, calorie and fat goals, physical activity goals, and guidelines for self-monitoring. All participants will receive training in core BWL strategies and techniques to help them achieve these goals, and personalized feedback on their progress throughout the program. The key differences between the arms are the explicit emphasis on facilitating autonomy, competence and relatedness in the BWL+Aut arm and the use of extrinsic rewards (financial incentives) coupled with loss and regret aversion messaging in the BWL+Ext arm.

8.1 Adapted Behavioral Weight Loss (BWL; aka "SILVER GROUP")

Consistent with standard BWL programs, this arm is grounded in learning theory and focuses on making changes in the environment to support healthy eating and activity. The importance of establishing rewards for new behaviors is emphasized to help reinforce the behavior and maintain it over time. Further, this arm is focused on teaching strategies (e.g., goal-setting, stimulus control) to help participants implement eating and activity prescriptions and achieve energy imbalance. Feedback and messaging provided is: tailored, directive, and based on progress toward program goals (based on data reported) and is consistent with standard BWL recommendations.

8.2 BWL + Autonomous Motivation (BWL+Aut; aka "GREEN GROUP")

The BWL+Aut arm maintains many of the core tenets of standard BWL (e.g., stimulus control, goal-setting, changing eating and activity behaviors to create energy imbalance). However, BWL+Aut draws heavily from Self-Determination Theory (SDT) and emphasizes the importance of autonomous self-regulation and integration of behavioral goals with personal values, with the aim of improving engagement and sustained behavior change. Feedback and messaging provided is: tailored, autonomy supportive, and based on progress toward goals (based on data reported) but framed to enhance autonomy and choice. Additional components include: optional experiential group sessions offered in the community (e.g., circuit training class) that are meant to promote both competence and relatedness with others. These sessions are optional and designed to help apply the lesson content taught, but new BWL content is not provided.

8.3 BWL + Extrinsic Motivation (BWL+Ext; aka "BLUE GROUP")

The BWL+Ext arm is consistent with the theoretical foundation of BWL and focuses on all core strategies described in the BWL program. In addition, this arm pays particular attention to the role of establishing extrinsic rewards; small financial incentives are provided for meeting engagement and weight loss targets. Drawing on principles of learning theory and behavioral economics, the financial rewards are offered frequently, varied in amount, and the schedule is unknown to participants. Further, loss and regret aversion messaging is used to amplify the effects of the modest financial incentives. Feedback and messaging provided is: tailored, directive, incorporates regret and loss aversion language, and based on progress toward program goals (based on data reported) and is consistent with standard BWL recommendations. Details of the incentive structure are as follows: 1) Guaranteed incentives linked to process / behavior; each week that participants self-monitor ≥4 days of weight and dietary intake they earn a variable incentive ranging from $1-10. The maximum amount that can be earned over the 6-months is $90. And 2) Lottery-based / not guaranteed incentives linked to outcome / weight loss; participants who lose 5-10% of weight are entered into a $50 raffle; those who lose >10% are entered into a $100 raffle. At each of the 3- and 6-month visits, 3 $50 winners and 3 $100 winners are drawn.

8.4. Contact Schedules

Participants in all arms will receive one 90-minute group-based session (Weight Loss 101 and intro to website) and one 45-minute individual session to personalize goals, followed by technology mediated program and weekly personalized feedback on their progress (pre-determined based on categories / algorithms weekly and human e-coaching monthly). All sessions and e-coaching will be conducted by a lifestyle coach with a background in BWL and training in the study protocol. New lesson content is pushed out via email once per week with unique links to specific components of the content for the week, and full lesson content is available on the website. Two text messages are delivered each week, one on Thursday and one on Sunday (motivational and reminder, respectively). Personalized feedback is made available on website and pushed to email once per week. Participants have the option to contact their coach with additional questions throughout the week; coaches are trained to fold responses into their feedback when possible.

8.5. Challenges

Participants in all arms will be invited to participate in a series of 2-week weight loss challenges designed to encourage the application of core behavioral weight loss principles. These challenges will occur at 4 points over the course of the intervention: weeks 1-2, 7-8, 13-14, and 19-20. Each challenge is grounded in self-regulation and designed to help participants practice this core skill. Over the course of each challenge, participants will be asked to self-weigh and then use the information on the scale to guide their behavior. If participants have experienced some weight loss they will be reminded to reward praise themselves and continue their efforts. Further, each challenge will have a predetermined behavioral strategy to assist participants if weight loss progress is not observed (e.g., decreasing red foods). The timeline, goal, content, and implementation of each challenge will be identical across groups; however, the reinforcement for meeting the weight loss goal varies as a function of treatment arm:

8.5.1. BWL: Participants will be encouraged to develop their own reward plan prior to beginning each challenge and enact that plan if they meet the challenge weight loss goal. Rewards will not be provided by the study; rather, each participant will select a reward that is personally salient with receipt contingent upon achieving the goal.

8.5.2. BWL+Aut: Participants who achieve the weight loss goal associated with each challenge will be entered into a raffle to win a skills-based prize to promote competence (e.g., resistance bands, steamer basket for food preparation, spiralizer). Participants will be able to choose their prize from several options in order to enhance autonomy and personal relevance. Up to 5 winners will be drawn for each challenge; entrance into raffle will be determined based on objective weight data received via participants' Bluetooth-connected scales over the 2-week challenge period.

8.5.3. BWL+Ext: Participants who achieve the weight loss goal associated with each challenge will be entered into a raffle to win $50. Up to 5 winners will be drawn for each challenge; entrance into raffle will be determined based on objective weight data received via participants' Bluetooth-connected scales over the 2-week challenge period.

8.6. Optional Closed Facebook Group

Given that participants are recruited in cohorts, but that the majority of intervention contact is conducted via a technology platform that is individual level, we want to offer participants the opportunity to interact with one another and receive social support. We have created “secret” or closed groups for each intervention arm and participants will have the option to join. Interventionists will post weekly (e.g., notifying participants that new content is available on the intervention website, challenges to help participants apply the material and promote engaging with one another), but no new intervention content will be delivered via Facebook. Weekly posts will consist of several types or categories including: messages encouraging participants to log in to the intervention platform and view new lesson for that week; messages to encourage and motivate participants as they engage in key behavior changes; messages pointing participants to helpful resources or materials available on the intervention platform; messages encouraging participants to support one another as they go through the program; and arm specific posts including announcements for experiential class offerings (BWL+Aut arm only) and incentives won in the previous week (BE arm only). Participants’ decision whether or not to join the closed Facebook group will not impact any other aspects of the intervention they will receive and is completely voluntary. The groups are closed and thus not searchable or viewable by anyone on Facebook unless they have received an invitation from our intervention team. These invitations will be limited to randomized participants who have already consented to participate in the trial and closed Facebook groups will be specific to treatment arm.

9. Data Collection

9.1 Assessments

All assessments will be completed by staff members who are blinded to the participants’ intervention assignment and have been trained and certified by the Principal Investigator and Research Coordinator by demonstrating competence in completing physical measurements. Participants will be provided a $50 honorarium for completing the 3- and 6-month assessments, and $75 for completing the 12-month assessment.

Measure	Timepoint							
	**Prescreen**	**Baseline**	**2 Weeks**	**2 Mos**	**3 Mos**	**4 Mos**	**6 Mos**	**12 Mos**
Anthropometric & Physical								
Weight (primary outcome)		X			X		X	X
Height		X			X		X	X
Waist Circumference		X			X		X	X
Body Composition		X			X		X	X
Resting Metabolic Rate		X					X	
Behavioral, Psychological & Environmental								
Disordered Eating (Eating Disorder Screen & Compensatory Behaviors)	X	X			X		X	X
AUDIT		X						
Alcohol Use		X					X	X
Weight Control Strategies Scale		X			X		X	X
Self-Weighing		X			X		X	X
Activity – objective (wrist bands)		X					X	X
Sleep		X					X	X
TSRQ		X	X	X	X	X	X	X
PCS		X	X	X	X	X	X	X
HCCQ		X	X	X	X	X	X	X
Intrinsic/Extrinsic Motivation		X	X	X	X	X	X	X
Built Environment		X					X	X
Dietary Intake		X					X	X
Eating-related Behaviors		X			X		X	
Day-to-Day Discrimination Scale		X					X	X
Weight Bias Internalization Scale		X					X	X
CES-D (depression)		X			X		X	X
Life Events		X					X	X
Perceived Stress Scale (PSS)		X			X		X	X
Difficulties in Emotion Regulation (DERS)		X					X	X
Brief Resilience Scale		X			X		X	
Resilience Scale for Adults		X			X		X	
Behavior Rating Inventory of Executive Function – Adult Version (BRIEF-A)		X					X	
Morningness-Eveningness Questionnaire (MEQ)		X						
Perception of Racial Discrimination Questionnaire		X						
Heightened Vigilance Scale—Abbreviated		X						
Loss of Control over Overeating Scale—Brief (LOCES-B)		X						
Multidimensional inventory of Black Identity (MIBI)		X						
Multidimensional Scale of Perceived Social Support		X						
Medical								
Blood Pressure		X			X		X	X
Medication Use		X			X		X	X
Medical Events		X			X		X	X
Other Questionnaires								
Demographics		X						
Financial Strain		X						
Contact Information		X						
Treatment Satisfaction/Feedback							X	
Adherence								
Attendance at in-person sessions		Throughout Intervention						
Weekly engagement data (weight days, diet days, log ins)		Throughout Intervention						

9.1.1. Data Collection Schedule

Shaded cells represent measures necessary to address primary and secondary study aims.

9.1.2 Anthropometric Measures

Weight, height and BMI. Weight will be measured in light clothes, without shoes, on calibrated scales. Two measures will be taken and if the difference exceeds 0.2 kg, a third will be taken. Height will be assessed with a wall-mounted stadiometer, using standard procedures. Two measures will be taken and if the difference exceeds 0.5 cm, a third will be taken. BMI will be calculated: weight in kg/height in m^2. (Weight and height will be measured at baseline, 3, 6 and 12 months)

Waist circumference**.** Waist circumference will be measured using the mid-point between the highest point of the iliac crest and the lowest part of the costal margin in the mid-axillary line using a Gulick tape measure. Two measures of waist circumference will be taken and the average of the measures will be used; if the difference of the two measures exceeds 0.5 cm, a third measure will be taken. (Waist circumference will be measured at baseline, 3, 6 and 12 months).

Body composition. Body composition will be assessed via bioelectrical impedance analysis (BIA) using the Tanita BC-418 Segmental Body Composition Analyzer. Participants will be asked to fast for 4 hours, refrain from alcohol for at least 12 hours, and to refrain from strenuous exercise or sauna for 8 hours prior to testing. (Body composition will be assessed at baseline, 3, 6 and 12 months).

Blood Pressure. Blood pressure will be assessed with a Dinamap Carescape V100. Cuff size will be determined by arm circumference. After a 5-minute rest period and with both feet flat on floor, three readings will be taken, with at least 30-seconds between measurements. (Blood pressure will be assessed at baseline, 3, 6 and 12 months).

Resting Metabolic Rate. Resting metabolic rate will be assessed with a Fitmate GS. Participants will be asked to fast for 12 hours, refrain from alcohol for at least 12 hours, refrain from smoking for 4 hours, and to refrain from strenuous exercise or sauna for 8 hours prior to testing. (Resting Metabolic Rate will be assessed at baseline and 6 months).

9.1.3. *Behavioral, Psychological and Environmental Measures*

AUDIT + Alcohol Use. The Alcohol Screen asks about recent alcohol intake whereas the AUDIT assesses whether a potential participant has current symptoms of alcohol dependence. These questions will be completed by the participant in clinic; with the exception of the skip on Question 1, each question must be answered. Individuals who score <8 on the AUDIT are eligible for participation and do not require additional follow up. If an individual scores between 9-15 on the AUDIT, a behavioral specialist or psychologist will review the form with the individual and provide an assessment of the individual's drinking behavior to Dr. LaRose; determination regarding eligibility will be made on an individual basis taking into account the full clinical picture. If an individual scores >16 on the AUDIT, they are ineligible for participation and the behavioral specialist should provide resources / referrals for relevant services. (AUDIT will be administered at Baseline, and Alcohol Use will be assessed at Baseline, 6 and 12 months).

Eating Disorder Screen. The EDS consists of 6 items, all of which are yes / no. Any "yes" response on item 6, or >2 abnormal responses overall will warrant follow up by the behavioral specialist to determine whether it is safe and appropriate for the individual to participate in this trial; recommendations will be made to Dr. LaRose and a determination regarding eligibility will be made on a case by case basis taking into account the full clinical picture. (EDS will be administered at Pre-screening, 3, 6 and 12 months).

Compensatory Behaviors. A series of items will be asked to assess whether participants have a history of engaging in disordered eating behaviors to avoid weight gain or induce weight loss (e.g., vomiting, laxative abuse). Participants will be asked whether they have ever engaged in these behaviors, and whether they have engaged in the behaviors within the previous 3 months. Affirmative responses will be reviewed with Dr. LaRose to determine whether it is safe for the individual to participate in the intervention. In addition, they will be asked at follow up assessments to determine whether any disordered eating behaviors have developed during the course of the intervention; all instances will be reviewed with the PI to determine whether it is safe for the

individual to continue participating in the intervention (These items will be administered at pre-screening, 3, 6 and 12 months).

CES-D (Depression Questionnaire): Depression will be assessed at screening using the 20-item CES-D self-report questionnaire. This is not a clinical tool, nor a diagnostic tool. However, any participant who scores >16 on the CES-D should receive follow up questions and discussion with a behavioral specialist as part of the behavioral interview to determine whether this program is appropriate for the participant. (CES-D will be administered at Baseline, 3, 6 and 12 months).

Self-Weighing. Frequent self-weighing is the pivotal component of self-regulation. To assess this, participants will be asked to indicate how frequently they have weighed themselves, ranging from several times a day, daily, a few times a week, weekly, once a month, or less than once a month to never. Participants will also be asked to indicate the extent to which they found weighing themselves to be frustrating or motivating and how they reacted if they observed weight gains. (Self-Weighing will be administered at Baseline, 3, 6 and 12 months)

Weight Control Strategies. The Weight Control Strategies Scale (WCSS) is a 30-item measure assessing the frequency with which respondents engage in strategies to control their weight. The WCSS contains four subscales—Self-Monitoring, Physical Activity, Psychological Coping, and Dietary Choices—all of which are targets of the current intervention. (Weight Control Strategies will be administered at Baseline, 3, 6 and 12 months)

Sleep. A questionnaire will be administered that asks about duration of sleep and problems encountered during sleep (e.g. snoring). The items are identical to those being used in the ongoing EARLY trials. 26 (Sleep Habits will be administered at Baseline, 6 and 12 months).

Dietary Intake Interview. Participants will respond to several interviewer-administered modules to assess dietary intake across core intervention targets (i.e., sugared beverages, fat, fast food, alcohol, fruit and vegetables). Also, participants will report relevant eating behaviors (e.g., breakfast, meals away from home). These items have been used in our previous studies with young adults and are taken in part from large-scale national health surveys (NHANES and BRFSS) and the NCI Percentage Energy from Fat Screener. The domains assessed in this interview are high-risk behaviors associated with overweight and obesity among young adults and are explicit intervention targets; thus, this measure will be administered at baseline, 6-month, and 12-month assessments in order to detect change.

Perceived Stress (PSS). The Cohen Perceived Stress Scale is a 4-item self-report instrument that captures the participant's perception of stress in their lives over the past month. The Perceived Stress Scale poses general questions about current stress levels. All items begin with the phrase: In the past month, how often have you felt…? This instrument has been used in many studies and has excellent reliability and validity. (Perceived Stress will be administered at baseline, 3, 6 and 12 months).

Physical activity and Objective Sleep. Physical activity (PA) and Sleep will be objectively assessed at each assessment using the previously validated Actigraph GT9X Link. This monitor is worn on the wrist and assesses movement and energy expenditure using a validated 3-axis accelerometer and data filtering technology. Minute-by-minute data will be analyzed to determine the amount of time spent in sedentary, light, moderate, and vigorous intensity activities. Participants will wear the device for 1 week at each assessment. During this time, they will record the time that they go to sleep and wake up. A minimum wear-time threshold (≥4 days of ≥8 hrs/day) must be met in order to be included in data analyses. (Physical Activity will be assessed at Baseline, 6 and 12 months).

Day-to-Day Discrimination Scale. This scale will be used to measure the frequency of experiences of weight discrimination from others. The scale was originally developed to examine prevalence rates of perceived discrimination as part of the large national MIDUS survey [54], and has been used to look specifically at perceived discrimination due to weight [55]. The scale was modified for an adolescent/young adult sample [56] and several additional questions related specifically to weight stereotypes have been added [57]. The scale shows consistently high internal validity (Cronbach alphas > .90) and predictive ability. The 18 item scale asks "how often on a day-to-day basis do you experience each of the following situations because of your body weight" on a (1) never to (4) often scale with such items as "You are treated with less courtesy than other people" and

"People don't want to get to know you better." (Day-to-Day Discrimination Scale will be administered at Baseline, 6 and 12 months)

Weight Bias Internalization Scale. The Weight Bias Internalization Scale (WBIS; [58]) will be used to measure the extent to which a participant believes that negative stereotypes and beliefs about body weight apply to herself or himself. Participants are asked their extent of agreement with statements such as "If only I had more willpower I wouldn't be the weight that I am" (1=strongly disagree to 7=strongly agree). This scale is a widely used metric for internalized weight bias, and the 10-item version (used in this study) has good internal consistency and construct validity [59-60]. (Weight Bias Internalization Scale will be administered at Baseline, 6 and 12 months).

Life Events. The life events questionnaire from the CARDIA study lists 67 events and participants are asked to indicate whether or not that event has occurred in the past year. We have chosen to use the CARDIA life events questionnaire in preference to other similar questionnaires because it was developed specifically for young adults and reflects the type of life events that occur most commonly in this age group. (Administered at Baseline, 6 and 12 months)

Difficulties in Emotion Regulation Scale. The Difficulties in Emotion Regulation Scale (DERS) will be used to assess 6 dimensions of emotion dysregulation: non-acceptance of emotional responses, difficulty engaging in goal-directed behavior, impulse control difficulties, lack of emotional awareness, limited access to emotion regulation strategies, and lack of emotional clarity. Respondents are asked to indicate on a scale from 1 (almost never) to 5 (almost always) how often they experience the items listed. (DERS will be administered at Baseline, 6 and 12 months).

Brief Resilience Scale. The Brief Resilience Scale (BRS) is a 6-item measure that assesses participants' individual-level global resilience, or ability to "bounce back" from stressful situations. Participants are asked to rate their agreement with each item on a 5-point scale ranging from "strongly disagree" to "strongly agree," with higher scores indicating greater resilience. (BRS will be administered at Baseline, 3-month, and 6-month assessments).

Resilience Scale for Adults. The Resilience Scale for Adults (RSA) is a 33-item measure that assesses participants' capacity for resilience across multiple domains including perception of self, perception of future, structured style, social competence, family cohesion, and social resources. Higher scores on this measure reflect greater resilience. (RSA will be administered at Baseline, 3-month, and 6-month assessments).

Behavior Rating Inventory of Executive Functioning in Adults (BRIEF-A). The BRIEF-A is a 75-item self-report questionnaire that assesses respondents' perceived everyday executive functioning across a variety of domains. This measure yields 10 clinical scale scores: Inhibit, Self-Monitor, Plan/Organize, Shift, Initiate, Task Monitor, Emotional Control, Working Memory, and Organization of Materials. These scales load onto 2 broad factors—Behavioral Regulation and Metacognition—and scores can also be summed to yield a global score. The BRIEF-A has embedded validity scales in order to maximize internal validity. Respondents' scores are compared to age-based norms in order to determine the clinical severity of reported symptoms measured against similarly-aged peers. (The BRIEF-A will be administered at Baseline and 6-month assessments).

Morningness-Eveningness Questionnaire (MEQ). The MEQ is a 19-item self-report questionnaire developed to categorize respondents into chronotypes based on their total score across all items. The MEQ was developed and validated in a sample of young adults is widely used as a measure of chronotype. MEQ scores are associated with biological indications of chronotype such as waking oral temperature as well as core body temperature and melatonin levels. (The MEQ will be administered at Baseline).

Perceptions of Racial Discrimination Questionnaire. Perceived racial discrimination will be assessed using the Perception of Racial Discrimination Questionnaire, which consists of 8 items that ask respondents about acts of racial discrimination experienced personally (e.g., I feel like I am personally a victim of society because of my race.) and within the context of the group (e.g., My racial group is discriminated against.) The questionnaire has excellent internal consistency ($\alpha = .84$). (Perceptions of Racial Discrimination Questionnaire will be administered at Baseline.

Heightened Vigilance Scale—Abbreviated. This is a 4-item questionnaire that asks respondents to assess the degree to which they prepare to be discriminated against because of their race. Participants respond on a 6-point Likert scale ranging from never to almost every day for each of the items. The heightened vigilance scale abbreviated has good reliability (α = .72). Administered at baseline. (Heightened Vigilance Scale—Abbreviated will be administered at Baseline.

Loss of Control Overeating Scale—Brief (LOCES-B). This 7-item questionnaire asks respondents to assess their eating behaviors over the past 4 weeks (28 days). The LOCES-B has excellent internal consistency (α = .93) and is highly correlated with the standard LOCES (r=.96). Participants respond on a 5-point Likert scale ranging from never to always. The LOCES-B is ideal for research purposes and assessing associations between eating disturbances and weight related outcomes. (LOCES-B will be administered at Baseline).

Multidimensional Inventory of Black Identity (MIBI)—Centrality and Regard subscales. The Centrality subscale of the MIBI consists of 8 items that ask respondents to assess the extent to which they believe being African American is central to their self-definition (e.g., "Being Black is unimportant to my sense of what kind of person I am."). Regard is made up of a public (e.g., "Blacks are not respected by the broader society.") regard and private (e.g., "I often regret that I am Black.") regard subscale. Each regard subscale consist of 6 items each. The MIBI subscales have good internal consistency (α =60 to .80) (MIBI subscales will be administered at Baseline).

Multidimensional Scale of Perceived Social Support. This 12-item questionnaire was designed to measure perceptions of support from family, friends, and significant others. Participants respond on a 7-point Likert scale. All subscales of the multidimensional scale of perceived social support have excellent internal consistency (Family α =.90; Friends α = .94 ; Significant Other α =.95). (Multidimensional Scale of Perceived Social Support will be administered at Baseline).

9.1.4. *Potential Mediators of Treatment Outcome*

Autonomous Self-Regulation. Autonomous self-regulation will be assessed using the Treatment Self-Regulation Questionnaire (TSRQ) [63-64]. The TSRQ asks individuals why they would try and control their weight and lists 12 different reasons. Half of the items reflect autonomous motivation (e.g., "Because I feel that I want to take responsibility for my own health") and half reflect controlled motivation (e.g., "Because I would feel guilty or ashamed of myself if I did not try to control my weight"). Participants are asked to respond on a 7-point scale ranging from "not at all true" to "very true" to indicate the extent to which each reason is true for them. The TSRQ has adequate reliability (α > .80)[63] and construct validity [64]. Previous studies have demonstrated a positive relationship between autonomous self-regulation and weight loss [42,64]. (Autonomous Self-Regulation (TSRQ) will be administered at Baseline, week 2, and months 2, 3, 4, 6 and 12)

Perceived Competence. Competence will be assessed using the Perceived Competence Scale (PCS) [65], a 4-item questionnaire that asks respondents to assess the degree to which they feel able to change or sustain a health behavior. Participants respond on a 7-point scale ranging from "strongly disagree" to "strongly agree" for each of the items. The PCS can be adapted to target different behaviors; in the proposed study it will target both healthy eating and physical activity. The PCS has strong internal consistency (α > .80) and discriminant validity.[40] The PCS will be administered to participants in the current study to evaluate their confidence over time with respect to creating a healthier lifestyle. Previous studies suggest that perceived competence might be a mechanism of change in the relationship between autonomy and positive health behaviors [65]. (Perceived Competence (PCS) will be administered at Baseline, week 2, and months 2, 3, 4, 6 and 12)

Autonomy Support. Autonomy support will be assessed using the Health Care Climate Questionnaire [49]. The HCCQ is a 15-item questionnaire designed to assess perceptions of autonomy support. Participants respond on a 7-point scale ranging from "strongly disagree" to "strongly agree". In this study, it will be used to assess to degree to which participants perceive the feedback they received via the website to be autonomy supportive. The HCCQ has demonstrated excellent internal consistency (α = .95) in a community adult population [49], and has been used in a variety of topic areas including weight loss, smoking cessation, and diabetes [49]. Previous research has demonstrated a positive relationship between perceived autonomy support and weight loss [49,64]. (Autonomy Support (HCCQ) will be administered at Baseline, week 2, and months 2, 3, 4, 6 and 12)

Intrinsic and Extrinsic Motivation. Intrinsic and extrinsic motivation for weight loss will be assessed using a modified, treatment self-regulation questionnaire. The measure has excellent internal consistency and scores are associated with intervention outcomes [49,64]. Dr. Leahey (Co-I) has added items to the extrinsic motivation subscale of this measure to include financial motivation and with these additions, reliability was maintained at good to excellent levels (intrinsic α=.79; extrinsic α=.99). Moreover, this measure has demonstrated validity in our trials; our financial incentive weight loss program yielded higher extrinsic motivation scores compared to our weight loss program without financial rewards (24.7 vs. 14.7, $p<.01$) and both intrinsic and extrinsic motivation were associated with weight loss outcomes ($r=.20$, $p=.011$; $r=.42$, $p<.001$) [66]. (Intrinsic and Extrinsic Motivation will be administered at Baseline, week 2, and months 2, 3, 4, 6 and 12)

9.1.5. *Other Measures*

Demographics. Basic demographic, health and weight loss history information will be obtained at *baseline only*.

Financial Strain. Since money is a commonly cited barrier to improving diet and exercise in this population, we will assess financial strain at *baseline only*. Participants will be asked to indicate how much money they receive from a variety of sources (e.g., employment, family/friends, loans) in addition to how difficult they find it to live on their total household income and how many people that income is expected to provide for/support.

Engagement. In all arms, we will track attendance at the initial group session, the number of log-ons, lessons viewed, and the number of days and weeks participants report on weight and key behaviors via website. In the BWL+Aut arm, we will also track the number of optional community-based experiential sessions attended.

Participant Satisfaction. *At 6 months only* participants will be asked a series of items used in our previous work to assess satisfaction, perceived quality of the interventions, and perceived relevance for this age group.

10. Data Management and Quality Control

10.1 Data Management

Questionnaire data will be collected via REDCap, a secure survey data collection system available through VCU. Participants will receive a link to complete their measures online 48 hours before their visit. The day of the visit, the (blinded) staff member will log on and review responses for completion; any missing data will be addressed during the visit. During the visit, staff will record physical measurements on a form developed for this purpose; this form will be accompanied by a checklist prompting the assessor to review that all pre-assessment conditions have been met. These data will be entered into a password protected data entry and storage system, developed by our data manager, which provides programmatic protection against invalid data value entries, and provides second-party, blinded double-entry data verification to validate accuracy of data entry. Dr. Fava will be responsible for data cleaning, conducting error checks and preliminary analyses of all data to ensure accuracy.

REACH has customized collection and reporting tools to help clinic staff manage data collection. It includes customized tracking (e.g. adverse event reporting, intervention tracking) and quality control monitoring tools. Clinic and participant management tools have been developed to promote scheduling, retention reminders, and safety tracking.

10.2 Quality Control

Quality assurance and quality control is of the upmost importance for the REACH trial. The validity and eventual acceptability of study results depend in part on maintaining data integrity, documenting dropouts, monitoring and assessing protocol adherence, and unbiased measurements. The quality control program includes reviews of questionnaires, and duplicate measurements for instrument based work (e.g., anthropometry, blood pressure). The goal of all quality control work is to maintain a high degree of data quality throughout the course of the study and to document study quality for publications.

Training of Assessment Staff. A detailed Manual of Operations serves as the study procedural handbook and contains detailed information describing every aspect of the study including thorough descriptions of procedures

for recruitment, clinical and lab measurements, data collection and handling, monitoring and follow-up. Staff will receive training and certification in the physical assessment measures required on this study. In addition to mastering procedures outlined in the manual of operations, staff will observe the PI, Research Coordinator, or other trained team member in completing the assessment measures through a series of mock assessment sessions. The RC will directly observe research staff when they initially begin to conduct assessment visits on their own until she is satisfied that they have exceeded expectations in ability to build rapport and complete physical measurements without error.

Training for Treatment Sessions and E-Coaching. Treatment manuals have been developed to ensure the in-person sessions are delivered as intended. Monthly e-coaching messages will be written by interventionists in accordance with predetermined themes and formats. Interventionists will be masters or doctoral level individuals, who have previous experience conducting BWL and will receive thorough training in study procedures. All in-person sessions will be audiotaped; data submitted via the website and e-coaching messages will be monitored. Dr. Bean (Co-I) will review audio, participant data on website, and e-coaching messages, and meet with intervention staff for supervision specific to participant progress and/or safety concerns; Dr. LaRose will meet with intervention staff to oversee implementation of e-coaching messages in order to preserve study manipulation between arms. Drs. LaRose and Bean will also meet regularly to review any issues that arise. This will allow Dr. LaRose to remain blinded to the treatment weights and participant outcomes over the course of the trial but still remain actively engaged in the implementation of the trial and ensure proper execution of manipulation.

***Treatment Fidelity*.** Consistent with established protocols [67] two independent raters will trained by Dr. LaRose in the protocol and will perform formal fidelity assessments of the in person sessions and e-coaching. They will review a random sample of 20% of all audio and e-coaching to assess adherence to the protocol and competence. A fidelity checklist will be modified from our previous work and used to rate each of the selected sessions. Rater agreement will be calculated. The automated nature of the web lessons and automated feedback ensures fidelity to those aspects of the program.

11. Participant Management and Safety

11.1 Introduction

The following sections define medical events, serious adverse events, and unanticipated events and the procedures that will be followed in the trial to reduce the risk of all such occurrences.

11.2 Definitions

Definitions are obtained from the “Guidance on Reviewing and Reporting Unanticipated Problems Involving Risks to Subjects or Others and Adverse Events Office for Human Research Protections (OHRP)” [http://www.hhs.gov/ohrp/policy/AdvEvntGuid.htm]. The requirements and processes of the National Institute of Diabetes and Digestive and Kidney Diseases are also implemented.

11.2.1 Medical events and serious adverse events

An adverse event is defined as any untoward or unfavorable medical occurrence in a human subject, including any abnormal sign (for example, abnormal physical exam or laboratory finding), symptom, or disease, temporally associated with the subject’s participation in the research, whether or not considered related to the subject’s participation in the research. Abnormal laboratory results will be considered adverse events if they are not refuted by a repeat test conducted to confirm the abnormality or if the abnormality is of a degree that requires active clinical management.

Medical events and symptoms will be collected and reported from the beginning of study-related procedures to the end of the study follow-up period for an individual participant. At each assessment visit, REACH staff will specifically query participants for medical events using the Medical Events form. Information on adverse events may also be reported to study staff during intervention contacts, as well as through telephone calls and emails, and will be recorded on the study interim event form. Adverse events will be followed until resolution,

stabilization, or until it is determined that the study participation is not the cause. If there are any positive responses on the Medical Events form, the form will be reviewed by the appropriate study personnel (e.g., safety officer, study clinician, etc.) to determine if a Serious Adverse Event Form should be completed.

Consistent with NIH guidelines, **serious adverse events** (SAEs) are adverse events that meet any of the following criteria: fatal or life-threatening, poses an immediate risk of death, result in significant or persistent disability that lasted at least 1 month and changed your life, requires an overnight stay in the hospital but NOT the emergency room, result in a congenital anomaly/birth defect, or are important medical events that investigators judge to represent significant hazards or harm to research subjects. Any adverse event that meets any of these criteria (e.g. results in hospitalization) will be documented and reported as a serious adverse event. The serious adverse event form will be completed by staff or investigators with the help of the participant who can provide information about the event.

11.2.2 Unanticipated Problem

An unanticipated problem is defined as any incident, experience, or outcome that meets all of the following criteria: 1) unexpected 2) related or possibly related to participation in the research; and 3) suggests that the research places subjects or others at a greater risk of harm than was previously known or recognized. According to OHRP regulations, an incident, experience, or outcome that meets the three criteria for an unanticipated problem generally will warrant consideration of substantive changes in the research protocol or informed consent process/document or other corrective actions in order to protect the safety, welfare, or rights of subjects or others. Only a small subset of adverse events will be unanticipated problems.

11.3. Reporting of Serious Adverse Events and Unanticipated Problems

Dr. Elissa Jelalian, an external faculty member who is not involved with the study, will serve as an independent reviewer / Data Safety Monitoring Officer (DSMO). Dr. Wickham (Co-I) will serve as an internal medical and safety advisor for the proposed trial, and will provide guidance regarding any medical or safety concerns that arise over the course of the proposed trial. Dr. Wickham will be unblinded and able to review outcomes and alerts over the course of the trial and work with Dr. LaRose and Dr. Jelalian to ensure safety of the participants in the trial. Adverse events reported during the course of the study will be tracked and documented by Dr. LaRose (PI) and reported to the Virginia Commonwealth University Institutional Review Board within one week from the time the staff is notified or made aware of the event. Further, Dr. LaRose (PI) will contact Dr. Wickham (Co-I / internal safety) immediately should any adverse events or potential safety concerns arise to seek consultation and ensure that these issues are handled appropriately. In addition, Dr. Jelalian will meet with Dr. LaRose every 6 months to review progress, preliminary data, and adverse events. Additionally, any adverse events will be communicated to the DSMO, VCU IRB, and NIH within one week from the time the staff is notified, and any serious adverse events will be communicated to the DSMO, VCU IRB, and NIH immediately (or as soon as the PI or study staff are made aware of the SAE). The DSMO will make recommendations regarding the progress of the study and will be able to report to the Institutional Review Board or Office of Research Administration should they have concerns about the conduct of this research.

11.4 Potential Risks to Study Participants

The risks of this investigation are considered to be minimal. Participants may not lose weight, but this is a risk with any weight loss program. They may engage in unsafe dietary practices or may experience discomfort or minor injuries from becoming more active. However, precautions will be taken to minimize these risks and the intervention being provided is evidence-based, core BWL information that if followed, should produce a safe rate of weight loss (i.e., 1-2lbs per week); and gradually increasing physical activity will be encouraged. Alternative treatments for overweight and obesity include diets with lower daily calorie recommendations, pharmacological interventions, and surgical procedures. These treatments are considered to have greater risks than the lifestyle programs proposed in the present application. No sensitive information (e.g., child abuse, violence, some infectious diseases) will be collected during the research. No diagnoses will be made by the research team, and the research team will not have access to participants' medical records.

11.4.1 Anticipated adverse events in young adults

Adverse events, particularly serious events, not related to study participation are expected to be uncommon in this study of lifestyle intervention in generally healthy young adults. Nevertheless, young adults in this age range do experience acute health conditions and physical trauma that could result in serious adverse events, including disability, hospitalization and even death. Common causes of serious adverse events and/or hospitalization in this age range include mental disorders, digestive system diseases, unintentional injuries, genitourinary diseases, respiratory diseases, musculoskeletal diseases, endocrine diseases, neoplasm/cancer, diseases of the heart, pregnancy and pregnancy-related complications, and infections. Births with congenital anomalies also occur although rates are rare. Other adverse events that occur in young adults include the development or worsening of eating disorders (e.g. bulimia nervosa), spontaneous and elective abortion, asthma exacerbation, and various injuries, The most common causes of death in this age range in the US are unintentional injuries (accidents, homicide), intentional injury (suicide), malignant neoplasm's, diseases of the heart, congenital malformations, HIV disease, pregnancy/childbirth/puerperium, cerebrovascular diseases, diabetes mellitus, influenza/pneumonia, chronic lower respiratory diseases, chronic liver disease, and septicemia.

11.4.2 Minimization of risks

Participants in all arms will be advised about safe weight loss practices including dietary change and beginning an exercise program. Participants will be advised to gradually increase exercise over time. Weight will be measured objectively at the initial group session, and reported via the secure intervention website weekly during the treatment program. Weight will also be measured objectively as each assessment visit (0, 3, 6 and 12 months). Dr. Bean (Co-I) will meet with the intervention team regularly for supervision, listen to audio recordings of Weight Loss 101 sessions, and review participant data on the website. She and Dr. LaRose (PI) will meet regularly to discuss any supervision or safety concerns that arise across arms, allowing Dr. LaRose to remain blinded to treatment assignment. In the event that weight loss progresses too rapidly or participants report unsafe practices, the PI / Co-I will instruct the interventionist to discuss these issues with the participant and encourage alternative, healthier practices. If participants continue to report potentially unsafe weight loss practices, they will be referred for treatment and intervention will be suspended; this will be reported to the internal Data Safety Officer (Dr. Wickham), as well as the external Data Safety Monitoring Officer (DSMO). Further, Drs. LaRose and Bean will contact Dr. Wickham immediately if any other adverse events or potential safety issues occur to seek consultation and ensure that these issues are handled appropriately. A faculty member not directly involved with this application will be recruited as an independent Data Safety Monitoring Officer, and that individual will monitor the effects of treatment and if treatment results in unanticipated untoward consequences, the appropriate action will be taken. In addition, all investigators and research staff working on the proposed trial will complete Human Subjects Certification training through CITI and documentation will be on file with the VCU IRB; refresher courses will be completed annually.

Table 1. Alert Values and Action Required

ALERT	ACTION
Blood Pressure	
SBP ≥ 140 and /or DBP ≥ 90 mmHg	Clinic staff will inform the participant at time of measurement. Participants will be advised to see their primary healthcare provider within 1 month for an assessment.
SBP ≥ 160 and /or DBP ≥ 100 mmHg	Clinic staff will inform the participant at time of measurement. Participants will be advised to see their primary healthcare provider within 1 week for an assessment.
SBP ≥ 180 and /or DBP ≥ 110 mmHg	Clinic staff will inform the participant at time of measurement. Participants will be advised to see their primary healthcare provider within 1-2 days or evaluated immediately depending on clinical situation or presence of other symptoms (e.g., headache, shortness of breath, chest pain should be evaluated immediately), based on consultation with the internal safety advisor, Dr. Wickham.
Hypotension<br>SBP <90	Clinic staff will inform participant at time of measurement and ask “are you feeling dizzy or lightheaded?” If yes, participant should be advised to see their primary healthcare provider within 1 month, or sooner depending on the clinical situation or presence of other symptoms (e.g., passing out [syncope]). All instances in which other symptoms are present will be reviewed with Dr. Wickham to determine appropriate course of action.
Heart Rate	
Heart rate > 110 bpm	Clinic staff will inform the participant at time of measurement. Participant with a heart rate between 110-119 and no symptoms of lightheadedness/dizziness, chest pain or shortness of breath will be asked to see their provider for an assessment within 1-2 weeks. The internal safety advisor, Dr. Wickham, will be contacted if participants have a heart rate ≥120 bpm to provide triage recommendations.

Safety and Adverse Events Protocol:

Safety management in REACH is intended to promote the safety of participants as it relates to study protocol. If at any time a medical event is reported, study staff will complete the Medical Events Form. If the medical event constitutes an SAE, the Serious Adverse Events form will be completed as well. In addition, the Medical Events Form will be routinely administered to all participants at 3, 6 and 12-month follow up assessments to assess for any and all potential adverse events during the course of the study. Forms shall be completed by trained clinic staff, and reviewed by the investigator and co-investigators. <u>Page 7-8 of the Safety Chapter of the Manual of Procedures outlines what constitutes a medical event, a Serious Adverse Event, and details appropriate reporting guidelines.</u>

<u>NOTE</u>: The PI shall be notified immediately of any adverse events, SAE’s or potentially expedited events. Dr. LaRose will immediately report to Drs. Wickham and Jelalian, as well as the VCU IRB. A determination will be made at that time whether the incident requires reporting to NIDDK. Any situation in which you are uncertain whether an incident requires reporting, err on the side of reporting to Dr. LaRose immediately.

12. Analyses Plans

Statistical Analysis. Primary Aim 1 will examine the primary study hypotheses at 6 months: 1) the BWL+Aut arm will achieve significantly greater weight loss than the Web arm; 2) the Web+Ext arm will achieve significantly greater weight loss than the Web arm; and 3) the BWL+Ext arm will achieve significantly greater weight loss than the BWL+Aut arm. Secondary Aim 1 will evaluate the relative efficacy of the BWL+Aut, BWL+Ext, and BWL groups on important ancillary variables at 6-months. We expect the BWL+Aut and BWL+Ext arms to both achieve greater improvements in these variables than the BWL arm, and that the BWL+Ext arm will outperform the BWL+Aut arm. Exploratory Aim 1 is concerned with *weight loss maintenance; we will examine weight change across all arms from post-treatment (6 months) to follow up (12 months) and compare all arms on* weight maintenance at 12 months. Exploratory Aim 2 will explore whether the proposed theory-based intervention mechanisms help to *mediate the relationship between intervention arm and weight loss outcomes*. Details regarding planned analyses, sample size and power are presented below.

Preliminary Analyses. Although randomization should equalize group characteristics, preliminary analyses will examine comparability of participants in each of the arms at baseline on demographic, weight history, psychosocial and behavioral characteristics using chi-square tests or analysis of variance (ANOVA), depending on the specific variable characteristics (categorical or continuous). We will examine the distributional properties of continuously-scaled variables to determine if normalizing transformations should be applied before conducting further analyses. If group differences are found for any variables, we will evaluate them and statistically control them in outcome analyses (e.g., employing them as covariates in a linear mixed model analysis to control for their effects on a weight change outcome).

Analyses for Primary Aim 1. For Aim 1, the primary outcome of interest will be differences in weight change across the BWL, BWL+*Aut and* BWL+*Ext* arms at 6 months. We have powered our primary analysis to examine change in kilograms using a more conservative ITT methodology with planned contrasts to examine weight change between each pair of treatment groups at the 6-month assessment. Specifically, we will conduct a longitudinal linear mixed model analysis across both 3- and 6-month outcomes, covariate-adjusted for baseline weight, using Proc MIXED in SAS 9.3, which allows a variety of longitudinal covariance structures to be modeled. This procedure will allow us to conduct the appropriate contrast tests on the change in weight at 6 months, and additionally we can compare within group effects across time. We will also conduct an additional, less conservative modeling procedure within Proc MIXED that accommodates missing values under the assumption of missing at random using all available data, and we will compare these results to the ITT analysis. To determine the sensitivity of our findings to any changes in height with a population that may experience growth over the study, we will conduct parallel analyses of changes in body mass index (BMI).

Analyses for Secondary Aim. We will examine differences across arms on secondary outcomes of interest at 6 months, including, change in blood pressure, waist circumference, body composition, activity (*change in total energy expenditure and minutes of moderate and high intensity activity*) and dietary intake (*consumption of fast food, fruits and vegetables, sugar sweetened beverages, and alcohol*). Analyses will be similar to the linear mixed model analysis approach proposed for the primary aims above.

Analyses for Exploratory Aim 1. The outcome of interest will be the differences in maintenance of weight loss across arms over the no-contact follow up period. We will extend the longitudinal linear mixed model analyses as outlined above to include 12-months, with primary planned comparisons to examine the change in weight outcomes from 6 to12 months. We will also examine change in kilograms and BMI as outlined above.

Analyses for Exploratory Aim 2. We will examine whether the treatment effects for weight change are mediated by proposed mechanisms including *autonomous motivation and extrinsic motivation.* There are many approaches to mediation analysis [68]; we will employ a Structural Equation Modeling (SEM) approach using Mplus Version 7 [69] for these analyses. Mplus accommodates the evaluation of complex multivariate models of mediation (multiple mediators with multiple variable indicators) across time, and includes statistical tests of both direct and indirect (mediated) effects. We will also evaluate a series of nested models to explore how the modeling process changes as the effect paths of specific mediators are removed from the overall model.

REFERENCES

1. Muyle TP, Park MJ, Nelson CD, Adams SH, Irwin CE, Brindis CD. Trends in adolescent and young adult health in the United States. *J Adoles Health.* 2009; 45(1): 8-24.
2. Merten MJ. Weight status continuity and change from adolescence to young adulthood: Examining disease and health risk conditions. *Obesity.* 2010;18(7):1423-1428.
3. Gokee-LaRose J, Gorin AA, Raynor HA, et al. Are standard behavioral weight loss programs effective for young adults? *Int Obes*. 2009;33(12):1374-1380.
4. Loria CM, Signore C, Arteaga S. The need for targeted weight control approaches in young women and men. *Am J Prev Med*. 2010;38(2):233-5.
5. Poobalan AS, Aucott LS, Precious E, Crombie IE, Smith WC. Weight loss interventions in young people (18 to 25 year olds): A systematic review. *Obes Rev.* 2010;11(8):580-592.
6. Gokee-LaRose J, Leahey TM, Weinberg BM, Kumar R, Wing RR. Young adults' performance in a low-intensity weight loss campaign. *Obesity (Silver Spring).* 2012;20(11):2314-2316.
7. Raynor HA. Evidence-based treatments for childhood obesity. In: Jelalian E, Steele RG, eds. *Handbook of Childhood and Adolescent Obesity*. New York, NY: Springer;2008:201-220.
8. Delamater AM, Jent JF, Moine CT, Rios J. Empirically supported treatment of overweight adolescents. *Handbook of Childhood and Adolescent Obesity*. New York, NY: Springer;2008:221-240.
9. LaRose JG, Wing RR. Lifestyle approaches to obesity treatment. In: Rios MS, Ordovas JM, Gutierrez Fuentes JA. *Obesity*. Barcelona, Espana: Elsevier; 2010:311-322.
10. Gokee-LaRose J, Gorin A, Wing RR. Behavioral self-regulation for weight loss in young adults: A randomized controlled trial. *Int J Behav Nutr Phys Act*. 2009;6(10). PMID# 19220909
11. LaRose JG, Tate DF, Gorin AA, Wing RR. Preventing weight gain in young adults: A randomized controlled pilot study. *Am J Prev Med*. 2010;39(1):63-68.
12. LaRose JG, Lanoye A, Blumenthal M, et al. Comparing three methods of intervention delivery within a behavioral weight loss program for young adults: A randomized controlled pilot study. Presented at the annual meeting of The Obesity Society, Atlanta, GA. 2013.
13. Ryan RM, Deci EL. Self-determination theory and the facilitation of intrinsic motivation, social development, and well-being. *Am Psychol*. 2000;55:68-78.
14. Deci EL, Ryan RM. The 'what' and 'why' of goal pursuits: Human needs and the self-determination of behavior. *Psychol Inq*. 2000;11:227-268.
15. Ryan RM, Deci EL. An overview of self-determination theory. In: Ryan RM, Deci EL, eds *Handbook of Self-Determination Research*. Rochester, NY: University of Rochester Press. 2002.
16. Volpp KG, John LK, Troxel AB, Norton L, Fassbender J, Loewenstein G. Financial incentive-based approaches for weight loss: A randomized trial. *JAMA*. 2008;300(22):2631-7.
17. Bickel WK, Vuchinich RE. Reframing health behavior change with behavioral economics. Mahwah, NJ: Lawrence Erlbaum. 2000.
18. Park MJ, Mulye TP, Adams SH, Brindis CD, Irwin CE. The health status of young adults in the United States, *J Adolesc Health*. 2006;39:305-317.
19. Center for Disease Control. Costs of obesity. http://www.cdc.gov/obesity/data/adult.html. Accessed August 10, 2011.
20. Arnett, JJ. Emerging adulthood: A theory of development from the late teens through the twenties. *Am Psychol*. 2000;55(5):469-480.
21. Arnett, JJ. *Emerging Adulthood: The Winding Road from the Late Teens through the Twenties*. New York, NY: Oxford University Press; 2004.
22. Nelson MC, Story M, Larson NI, Neumark-Sztainer D, Lytle LA. Emerging adulthood and college-aged youth: An overlooked age for weight-related behavior change. *Obesity*, 2008; 16(10): 2205-11.
23. Gordon-Larsen P, Nelson MC, Popkin BM. Longitudinal physical activity and sedentary behavior trends: Adolescence to adulthood. *Am J Prev Med.* 2004;27(4):277–283.
24. Sidney S, Sternfeld B, Haskell WL, et al. Television viewing and cardiovascular risk factors in young adults: The CARDIA study. *Ann Epidemiol.* 1996;6(2):154–159.
25. Duffey KJ, Gordon-Larsen P, Jacobs DR, et al. Differential associations of fast food and restaurant food consumption with 3-y change in body mass index: The Coronary Artery Risk Development in Young Adults Study. *Am J Clin Nutr.* 2007;85(1): 201–208.

26. Niemeier HM, Raynor HA, Lloyd-Richardson E, et al. Fast food consumption and breakfast skipping: Predictors of weight gain from adolescence to adulthood in a nationally representative sample. *J Adoles Health.* 2006;39(6):842–849.
27. Huffman L, West DS. Readiness to change sugar sweetened beverage intake among college students. *Eat Behav.* 2007;8(1):10–14.
28. Arnett JJ, Schwab J. The Clark University poll of emerging adults: Thriving, struggling, & hopeful. 2012. Available at: https://www.clarku.edu/clark-poll-emerging-adults/pdfs/clark-university-poll-emerging-adults-findings.pdf.
29. Karg RS, Bose J, Batts KR, et al. Past year mental disorders among adults in the United States: Results from the 2008–2012 mental health surveillance study (CBHSQ data review). 2014. Available at: https://www.samhsa.gov/data/sites/default/files/NSDUH-DR-N2MentalDis-2014-1/Web/NSDUH-DR-N2MentalDis-2014.htm.
30. Wing RR. Behavioral approaches to the treatment of obesity. In: Bray GA, Bouchard C, eds. *Handbook of Obesity: Clinical Applications*. 2nd ed. New York, NY: Informa Healthcare; 2004:147-167.
31. Diabetes Prevention Program Research Group. Reduction in the incidence of type 2 diabetes with lifestyle intervention or metformin. *N Engl J Med*. 2002;346(6):393-403.
32. Lindstrom J. Sustained reduction in the incidence of type 2 diabetes by lifestyle intervention: Follow-up of the Finnish Diabetes Prevention Study. *Lancet*. 2006;368:1673-1679.
33. Look AHEAD Research Group. Reduction in weight and cardiovascular disease risk factors in individuals with type 2 diabetes: One year results of the look AHEAD trial. *Diabetes Care*. 2007;30(6):1374-1383.
34. Katterman SN, Butryn ML, Hood MM, Lowe MR. Daily weight monitoring as a method of weight gain prevention in healthy weight and overweight young adult women. *J Health Psychol*. 2015. Epub ahead of print. doi: 10.1177/1359105315589446.
35. West DS, Monroe CM, Turner-McGrievy G, et al. A technology-mediated behavioral weight gain prevention intervention for college students: Controlled, quasi-experimental study. *J Med Internet Res.* 2016; 18(6): e133. doi: 10.2196/jmir.5474.
36. Laska MN, Lytle LA, Nanney MS, Moe SG, Linde JA, Hannan PJ. Results of a 2-year randomized, controlled obesity prevention trial: Effects on diet, activity and sleep behaviors in an at-risk young adult population. *Prev Med.* 2016; 89: 230-236. doi: 10.1016/j.ypmed.2016.06.001.
37. Napolitano MA, Hayes S, Bennett GG, et al. Using Facebook and text messaging to deliver a weight loss program to college students. *Obesity.* 2013; 21(1): 25-31.
38. LaRose JG, Tate DF, Lanoye A, et al. Adapting evidence-based behavioral weight loss programs for emerging adults: A pilot randomized controlled trial. *J Health Psychol.* Forthcoming.
39. Lytle LA, Svetkey LP, Patrick K, et al. The EARLY trials: A consortium of studies targeting weight control in young adults. *Transl Behav Med*. 2014;3:304–313.
40. LaRose JG, Morrow Guthrie K, Lanoye A, et al. A mixed methods approach to improving recruitment and engagement of emerging adults in behavioral weight loss programs. *Obes Sci Prace*. 2016. Epub ahead of print.
41. Gorin AA. Autonomy support, self-regulation, and weight loss. *Health Psychol.* 2014:33(4):332-9.
42. Teixeira PJ, Silva MN, Jutta M, Palmeira AL Marland D. Motivation, self-determination, and long-term weight control. *Int J Behav Nutr Phys Act.* 2012;9:22.
43. Wing RR, Tate DF, Espeland MA, et al. Innovative self-regulation strategies reduce weight gain in young adults: The Study of Novel Approaches to Weight Gain Prevention (SNAP) randomized controlled trial. *JAMA Intern Med.* 2016; epub ahead of print, May 2.
44. Wing RR, Tate DF, Gorin AA, Raynor HA, Fava JL. A self-regulation program for maintenance of weight loss. *N Engl J Med*. 2006;355(15):1563-71.
45. Jeffery R, Bjornson-Benson WM, Rosenthal BS, et al. Effectiveness of monetary contracts with two repayment schedules on weight reduciton in men and women from self-referred and population samples. *Behav Ther.* 1984;15:273-9.
46. John LK, Loewenstein G, Troxel AB, et al. Financial incentives for extended weight loss: A randomized, controlled trial. *J Gen Intern Med.* 2011;26(6):621-626.
47. Leahey TM, Subak LL, Fava J, et al. Benefits of adding small financial incentives or optional group meetings to a web-based statewide obesity initiative. *Obesity.* 2015;23(1):70-76.
48. Towers Watson, National Business Group on Health. The road ahead: Shaping health care strategy in a post-reform environment. http://www.thehortongroup.com/Insurance_Library/The_Road_Ahead_Shaping_Health_Care_Strategy_ in_a_PostReform_Environment/. Accessed March 29, 2013.

49. Williams GC, McGregor HA, Sharp D, et al. Testing a self-determination theory intervention for motivating tobacco cessation: Supporting autonomy and competence in a clinical trial. *Health Psychol.* 2006;25(1):91-101.
50. Subak LL, Wing R, West DS, et al. Weight loss to treat urinary incontinence in overweight and obese women. *N Engl J Med.* 2009; 360: 481-490.
51. West DS, DiLillo V, Bursac Z, et al. Motivational Interviewing Improves Weight Loss in Women with Type 2 Diabetes. *Diabetes Care.* 2007;30:1081-1087.
52. Cohen J. *Statistical Power Analysis for the Behavioral Sciences*, 2nd Edition. Hillside, NJ: Lawrence Erlbaum; 1988.
53. Tate DF, LaRose JG, Wing RR. Utilization of different technologies to promote long term reporting and engagement in a weight gain prevention intervention targeting young adults: The Study of Novel Approaches to Prevention (SNAP). Submitted for presentation at the annual meeting of the International Society for Research on Internet Interventions. 2013;Chicago, IL.
54. Kessler R, Mickelson K, Williams D. The prevalence, distribution, and mental health correlates of perceived discrimination in the United States. *J Health Soc Behav*. 1999;40:208–230.
55. Carr D, Friedman MA. Is obesity stigmatizing? Body weight, perceived discrimination, and psychological well-being in the United States. *J Health Soc Behav.* 2005;46:244-259.
56. Quinn DM, Chaudoir SR. Living with a concealable stigmatized identity: The impact of anticipated stigma, centrality, salience, and cultural stigma on psychological distress and health. *J Pers Soc Psychol.* 2009;97:634-651.
57. Quinn DM, Puhl RM. National survey on weight discrimination, weight background, mental health, and weight maintenance. *Unpublished manuscript*. 2016;University of Connecticut
58. Durso LE, Latner JD. Understanding self-directed stigma: Development of the weight bias internalization scale. *Obesity(Silver Spring).* 2008;16:S80-S86.
59. Lee MS, Dedrick RF. Weight Bias Internalization Scale: Psychometric properties using alternative weight status classification approaches. *Body Image*. 2016;17:25-29.
60. Pearl RL, Puhl RM. Measuring internalized weight attitudes across body weight categories: Validation of the modified Weight Bias Internalization Scale. *Body Image.* 2014;11:89-92.
61. Stunkard AJ, Messick S. The three-factor eating questionnaire to measure dietary restraint, disinhibition and hunger. *J Psychosom Res.* 1985;29(1):71-83.
62. Kennardy J, Butler A, Carter C, Moor S. Eating, mood, and gender in a noneating disorder population. *Eat Behav.* 2003;4:149-158.
63. Levesque CS, Williams GC, Elliot D, Pickering MA, Bodenhamer B, Finley PJ. Validating the theoretical structure of the Treatment Self-Regulation Questionnaire (TSRQ) across three different health behaviors. *Health Educ Res*. 2007;22(5):691-702.
64. Williams GC, Grow VM, Freedman ZR, Ryan RM, Deci EL. Motivational predictors of weight loss and weight loss maintenance. *J Pers Soc Psychol*. 1996;70(1):115-126.
65. Williams GC, Freedman ZR, Deci EL. Supporting autonomy to motivate glucose control in patients with diabetes. *Diabetes Care*. 1998;21:1644-1651.
66. Leahey TM, LaRose JG, Wing RR. Effects of modest financial incentives on intrinsic and extrinsic motivation for weight loss. Accepted for presentation at the annual meeting of the Society of Behavioral Medicine;2014:Philadelphia, PA.
67. Bellg AJ, Borrelli B, Resnick B, et al. Enhancing treatment fidelity in health behavior change studies: Best practices and recommendations from the NIH Behavior Change Consortium. *Health Psychol*. 2004;23(5):443-451.
68. MacKinnon DP, Lockwood CM, Hoffman JM, West SG, Sheets V. A comparison of methods to test mediation and other intervening variable effects. *Psychol Methods*, 2002;7:83-104.
69. Muthén & Muthén, 1998-2013 Mplus User's Guide. Seventh Edition. Los Angeles, CA.
