# Supplementary material for: Effect of a Lifestyle Intervention on Cardiometabolic Health Among Emerging Adults: A Randomized Clinical Trial
Source: JAMA Netw Open. 2022 Sep 19;5(9):e2231903. doi: 10.1001/jamanetworkopen.2022.31903 (PMC9486452; doi:10.1001/jamanetworkopen.2022.31903)
Supplement: Supplement 2. — Data Sharing Statement [file jamanetwopen-e2231903-s002.pdf]

Data Sharing Statement

LaRose. Effect of a Lifestyle Intervention on Cardiometabolic Health Among Emerging Adults. *JAMA Netw Open*. Published September 19, 2022. doi:10.1001/jamanetworkopen.2022.31903

Data
Data available: Yes
Data types: Deidentified participant data, Data dictionary
How to access data: Requests for data can be made to the corresponding author (JGL) following publication of all planned primary and secondary papers by the investigative team. Any requests for data sharing must be reviewed and approved by the PI prior to the release of data. All data-sharing procedures would follow institutional and IRB policy at Virginia Commonwealth University, NIH policy, HIPAA and other local, state, and Federal laws and regulations.
When available: beginning date: 08-01-2024

Supporting Documents
Document types: None

Additional Information
Who can access the data: Requests for data sharing can be made to the corresponding author (JGL) following publication of all planned primary and secondary papers by the investigative team. Any requests for data sharing must be reviewed and approved by the PI prior to the release of data. All data-sharing procedures would follow institutional and IRB policy at Virginia Commonwealth University, NIH policy, HIPAA and other local, state, and Federal laws and regulations.
Types of analyses: For any purposes
Mechanisms of data availability: after approval of a proposal and with a signed data access agreement
